# A missense mutation in the MLKL brace region promotes lethal neonatal inflammation and hematopoietic dysfunction

**DOI:** 10.1038/s41467-020-16819-z

**Published:** 2020-06-19

**Authors:** Joanne M. Hildebrand, Maria Kauppi, Ian J. Majewski, Zikou Liu, Allison J. Cox, Sanae Miyake, Emma J. Petrie, Michael A. Silk, Zhixiu Li, Maria C. Tanzer, Gabriela Brumatti, Samuel N. Young, Cathrine Hall, Sarah E. Garnish, Jason Corbin, Michael D. Stutz, Ladina Di Rago, Pradnya Gangatirkar, Emma C. Josefsson, Kristin Rigbye, Holly Anderton, James A. Rickard, Anne Tripaydonis, Julie Sheridan, Thomas S. Scerri, Victoria E. Jackson, Peter E. Czabotar, Jian-Guo Zhang, Leila Varghese, Cody C. Allison, Marc Pellegrini, Gillian M. Tannahill, Esme C. Hatchell, Tracy A. Willson, Dina Stockwell, Carolyn A. de Graaf, Janelle Collinge, Adrienne Hilton, Natasha Silke, Sukhdeep K. Spall, Diep Chau, Vicki Athanasopoulos, Donald Metcalf, Ronald M. Laxer, Alexander G. Bassuk, Benjamin W. Darbro, Maria A. Fiatarone Singh, Nicole Vlahovich, David Hughes, Maria Kozlovskaia, David B. Ascher, Klaus Warnatz, Nils Venhoff, Jens Thiel, Christine Biben, Stefan Blum, John Reveille, Michael S. Hildebrand, Carola G. Vinuesa, Pamela McCombe, Matthew A. Brown, Benjamin T. Kile, Catriona McLean, Melanie Bahlo, Seth L. Masters, Hiroyasu Nakano, Polly J. Ferguson, James M. Murphy, Warren S. Alexander, John Silke

**Affiliations:** 1 The Walter and Eliza Hall Institute of Medical Research, Parkville, VIC 3052 Australia; 2 Department of Medical Biology, University of Melbourne, Parkville, VIC 3052 Australia; 3 Stead Family Department of Pediatrics, University of Iowa Carver College of Medicine, Iowa City, IA 52242 USA; 4 Department of Biochemistry, Toho University School of Medicine, Ota-ku, Tokyo, 143-8540 Japan; 5 Department of Biochemistry and Molecular Biology, Bio21 Institute, University of Melbourne, Melbourne, VIC 3052 Australia; 6 Computational Biology and Clinical Informatics, Baker Heart and Diabetes Institute, Melbourne, VIC Australia; 7 Translational Genomics Group, Institute of Health and Biomedical Innovation, School of Biomedical Sciences, Queensland University of Technology (QUT) at Translational Research Institute, Brisbane, Australia; 8 Department of Proteomics and Signal Transduction, Max Planck Institute of Biochemistry, Martinsried, 82152 Germany; 9 Vaccine and Gene Therapy Institute, Oregon Health and Science University, Beaverton, OR 97006 USA; 10 Victorian Clinical Genetics Services, Murdoch Children’s Research Institute, Parkville, VIC 3052 Australia; 11 The Royal Melbourne Hospital, Melbourne, VIC 3050 Australia; 12 Ludwig Institute for Cancer Research and de Duve Institute, Brussels, Belgium; 13 GSK Medicines Research Centre, Stevenage, UK; 14 CSL Limited, Parkville, VIC 3052 Australia; 15 Department of Immunology and Infectious Disease and Centre for Personalised Immunology (NHMRC Centre for Research Excellence), John Curtin School of Medical Research, Australian National University, Canberra, Australia; 16 Centre for Personalised Immunology (CACPI), Shanghai Renji Hospital, Shanghai Jiao Tong University, Shanghai, China; 17 Division of Rheumatology, The Hospital for Sick Children and the University of Toronto, Toronto, ON Canada; 18 Department of Neurology, University of Iowa Carver College of Medicine and the Iowa Neuroscience Institute, Iowa City, IA USA; 19 Faculty of Health Sciences and Sydney Medical School, University of Sydney, Sydney, Australia; 20 Department of Sports Medicine, Australian Institute of Sport, Bruce, ACT Australia; 21 Faculty of Health, University of Canberra, Canberra, Australia; 22 Department of Internal Medicine, Clinic for Rheumatology and Clinical Immunology, Medical Center –University of Freiburg, Faculty of Medicine, Freiburg, 79106 Germany; 23 Center for Chronic Immunodeficiency, Medical Center –University of Freiburg, Faculty of Medicine, Freiburg, Germany; 24 Princess Alexandra Hospital, Brisbane, QLD Australia; 25 Memorial Hermann Texas Medical Centre, Houston, TX USA; 26 Epilepsy Research Centre, Department of Medicine, University of Melbourne, Austin Health, Heidelberg, VIC 3084 Australia; 27 Murdoch Children’s Research Institute, Royal Children’s Hospital, Parkville, VIC 3052 Australia; 28 The University of Queensland, UQ Centre for Clinical Research, Royal Brisbane & Women’s Hospital, Brisbane, Australia; 29 NIHR Biomedical Research Centre, Kings College, London, UK; 30 Faculty of Health and Medical Sciences, The University of Adelaide, Adelaide, SA Australia; 31 Department of Anatomical Pathology, The Alfred Hospital, Prahran, VIC 3181 Australia

**Keywords:** Necroptosis, Inflammatory diseases, Haematopoietic stem cells

## Abstract

MLKL is the essential effector of necroptosis, a form of programmed lytic cell death. We have isolated a mouse strain with a single missense mutation, *Mlkl*^*D139V*^, that alters the two-helix ‘brace’ that connects the killer four-helix bundle and regulatory pseudokinase domains. This confers constitutive, RIPK3 independent killing activity to MLKL. Homozygous mutant mice develop lethal postnatal inflammation of the salivary glands and mediastinum. The normal embryonic development of *Mlkl*^*D139V*^ homozygotes until birth, and the absence of any overt phenotype in heterozygotes provides important in vivo precedent for the capacity of cells to clear activated MLKL. These observations offer an important insight into the potential disease-modulating roles of three common human *MLKL* polymorphisms that encode amino acid substitutions within or adjacent to the brace region. Compound heterozygosity of these variants is found at up to 12-fold the expected frequency in patients that suffer from a pediatric autoinflammatory disease, chronic recurrent multifocal osteomyelitis (CRMO).

## Abstract (web summary)

Necroptosis is a regulated form of inflammatory cell death driven by activated MLKL. Here, the authors identify a mutation in the brace region that confers constitutive activation, leading to lethal inflammation in homozygous mutant mice and providing insight into human mutations in this region.

## Introduction

Necroptosis is a lytic form of programmed cell death associated with the production of pro-inflammatory cytokines, the destruction of biological membranes and the release of intracellular damage associated molecular patterns (DAMPs)^1^. Necroptosis depends on the activation of the mixed lineage kinase domain-like (MLKL) pseudokinase by receptor interacting protein kinase 3 (RIPK3)^2–4^. RIPK3-mediated phosphorylation of MLKL triggers a conformational change^4,5^ that facilitates the translocation to, and eventual irreversible disruption of, cellular membranes. While the precise biophysical mechanism of membrane disruption is still a matter of debate, common features of contemporary models are the formation of an MLKL oligomer and the direct association of the executioner four-helix bundle domain (4HB) of MLKL with biological membranes^6–10^. In mouse cells, the expression of the murine MLKL 4HB domain alone (residues 1–125), 4HB plus brace helices (1–180), or the expression of phosphomimetic or other single site pseudokinase domain (PsKD) mutants is sufficient to induce membrane translocation, oligomerization and membrane destruction^4,9^. While capable of disrupting synthetic liposomes when produced recombinantly, similarly truncated and equivalent single site (PsKD) mutant forms of human MLKL do not robustly induce membrane-associated oligomerization and cell death without forced dimerization^11–13^. Furthermore, both mouse and human MLKL mutants have been reported that have the capacity to form membrane-associated oligomers, but fail to cause irreversible membrane disruption and cell death^9,13^. Recent studies have revealed that necroptosis downstream of MLKL phosphorylation and membrane association can be modulated by processes that engage the endosomal sorting complex required for transport (ESCRT) family of proteins. One model proposes a role for ESCRT in limiting necroptosis via plasma membrane excision and repair^14^ while other models limit plasma membrane disruption by ESCRT-mediated release of phosphorylated MLKL in extracellular vesicles^15–17^ and/or the internalization of phosphorylated MLKL for lysosomal degradation^17^.

In mice, the absence of MLKL does not appear to have obvious deleterious developmental or homeostatic effects^4,18^. However, genetic deletion of *Fadd*, *Casp8* or *Ripk1*, leads to inappropriate activation of MLKL and ensuing necroptosis during embryogenesis that is incompatible with life beyond embryonic day (E)10.5, E10.5 and 1–3 days post-natally, respectively^19–25^. Exploring the precise physiological consequences of inappropriate MLKL activation in these scenarios is complicated by the fact that FADD, Caspase-8 and RIPK1 also play important roles in cellular processes other than modulation of MLKL-induced necroptotic cell death^23,26–30^.

Aberrant levels of MLKL-dependent cell death contribute to disease in several genetic and experimental mouse models^23,31–35^. In humans, *MLKL* mRNA and protein levels are positively correlated with survival of patients with pancreatic adenocarcinoma, cervical-, gastric-, ovarian- and colon- cancers (reviewed by ref. ^36^). Interestingly, high levels of phosphorylated MLKL are associated with reduced survival in esophageal and colon cancer patients^37^. Two missense *MLKL* somatic mutations identified in human cancer tissue have been found to confer a reduction in necroptotic function in cell-based assays^4,13^. Very recently, siblings suffering from a novel neurodegenerative disorder were reported as homozygous for a rare haplotype involving a frameshift variant in *MLKL*, as well as an in-frame deletion of one amino acid in the adjacent fatty acid 2-hydroxylase (*FA2H*) gene^38^. The significant enrichment of an ultra-rare *MLKL* stop-gain gene variant p.Q48X has been reported in Hong Kong Chinese patients suffering from a form of Alzheimer’s disease^39^, however more common germline *MLKL* gene variants are only weakly associated with human disease in GWAS databases.

We have identified a single base pair germline mutation of mouse *Mlkl* that encodes a missense substitution within the MLKL brace region and confers constitutive activation independent of upstream necroptotic stimuli. Given this mutant *Mlkl* allele is subject to the same developmental and environmental controls on gene expression as wild-type *Mlkl*, the postnatal lethality in these mice provides insight into the physiological and pathological consequences of dysregulated necroptosis. In parallel, these findings inform the potential functional significance of three common human *MLKL* polymorphisms that encode non-conservative amino acid substitutions within, or in close proximity to, the brace helix that is mutated in the *Mlkl*^*D139V*^ mouse.

## Results

### Generation of a constitutively active form of MLKL

**Fig. 1:**
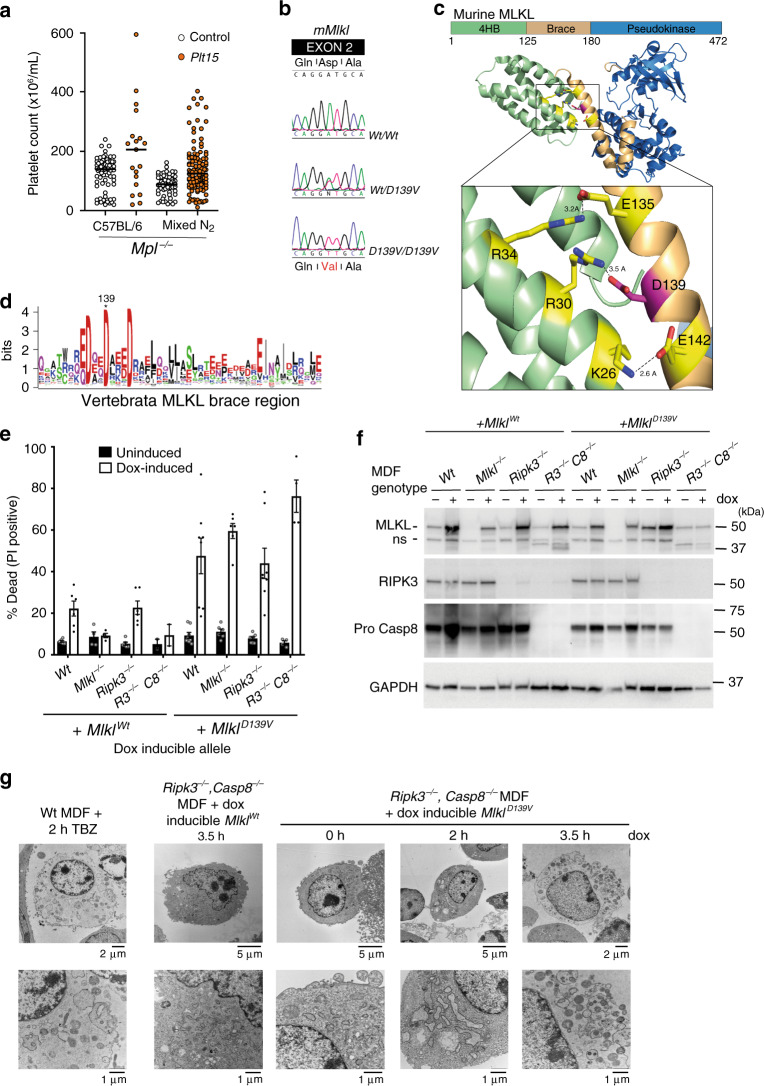
Murine MLKL^D139V^ is a constitutively active form of MLKL. **a** Platelet counts from *Mpl*^−/−^ mice (open circles, *n* = 80, 60) and offspring from matings between *Plt15* mice and *Mpl*^−/−^ mice (closed orange circles, *n* = 19, 113) on a C57BL/6 or mixed C57BL/6:129/Sv background used for linkage analysis (Mixed N_2_). **b** A missense mutation (D139V) in the second exon of *Mlkl* was identified in *Plt15* mutant mice. DNA sequence shown for wild type (top), a heterozygous mutant (middle), and a homozygous mutant (bottom). **c** Aspartate 139 contributes to an ‘electrostatic zipper’ joining brace helix 1 and the 4HB α2 helix of mouse MLKL (PDB code 4BTF)^4^. **d** Sequence logo of MLKL brace domain generated from multiple sequence alignment of all Vertebrata MLKL sequences (257) available on OrthoDB. **e** Mouse dermal fibroblasts (MDFs) of indicated genotypes were stably transduced with *Mlkl*^*Wt*^ and *Mlkl*^*D139V*^ and expression induced with doxycycline (dox, white bars) or not induced (black bars) for 21 h. PI-positive cells were quantified by flow cytometry. Means ± SEM are plotted for *n* = 4–8 experiments (a combination of biological repeats and independent experiments) for each genotype with the exception of *R3*^*−/−*^*C8*^−/−^ + *Mlkl*^*Wt*^ (*n* = 2, ±range). **f** Western blot analysis of whole cell lysates taken 6 h post doxycycline induction. **g** Transmission electron micrographs of MDFs stimulated as indicated. Images selected for (**f**) and (**g**) are representative of 2–3 independent analyses with similar results. TBZ; TNF + Birinapant + Z-VAD-fmk.

*Mpl*^−/−^ mice, owing to genetic deletion of the major receptor for thrombopoietin, have only 10% the wild-type number of peripheral platelets. An ENU mutagenesis screen was performed to identify mutations that ameliorate thrombocytopenia in *Mpl*^−/−^ mice via thrombopoietin independent platelet production^40^. A G_1_ founder, designated *Plt15*, had a modestly elevated platelet count of 189 × 10^6^ per mL compared with the mean for *Mpl*^−/−^ animals (113 ± 57 × 10^6^ per mL) and yielded 19 *Mpl*^*−/−*^ progeny. Ten of these mice had platelet counts over 200 × 10^6^ per mL, consistent with segregation of a dominantly acting mutation (Fig. 1a). Linkage analysis and sequencing identified an A to T transversion in *Mlkl* that was heterozygous in all mice with an elevated platelet count (Fig. 1b). The *Mlkl*^*Plt15*^ mutation results in a non-conservative aspartic acid-to-valine substitution at position 139 within the first brace helix. In the full-length mMLKL structure, D139 forms a salt bridge with an arginine residue at position 30 (α2 helix) of the MLKL four-helix bundle (4HB) domain^4^ (Fig. 1c). This salt bridge represents one of a series of electrostatic interactions between residues in helix α2 of the MLKL 4HB domain and the two-helix ‘brace’ region. D139 of mouse MLKL is conserved in all MLKL orthologues in vertebrata reported to date (Fig. 1d). We have shown that the exogenous expression of the 4HB domain of murine MLKL alone is sufficient to kill mouse fibroblasts whereas exogenous expression of full-length MLKL does not, suggesting an important role for this ‘electrostatic zipper’ in suppressing the killing activity of the MLKL 4HB^9^. To determine if MLKL^D139V^ exhibited altered ability to induce necroptotic cell death relative to MLKL^WT^, we stably expressed these full-length proteins under the control of a doxycycline-inducible promoter in immortalized mouse dermal fibroblasts (MDF) isolated from *Wt, Mlkl*^−/−^*, Ripk3*^*−/−*^ or *Ripk3*^−/−^*;Casp8*^*−/−*^ mice. While expressed at comparable levels, MLKL^D139V^ induced markedly more death than MLKL^Wt^, on each of the genetic backgrounds tested (Fig. 1e–f, Supplementary Fig. 1a), and formed a high molecular weight complex observable by BN-PAGE in the absence of exogenous necroptotic stimuli (Supplementary Fig. 1b). This indicates that MLKL^D139V^ is a constitutively active form of MLKL, capable of inducing necroptotic cell death independent of upstream signaling and phosphorylation by its activator RIPK3. Consistent with this interpretation, exogenous expression of MLKL^D139V^ in *Ripk3*^*−/−*^*;Casp8*^*−/−*^ MDFs was sufficient to induce the organelle swelling and plasma membrane rupture characteristic of TNF-induced necroptosis when examined by Transmission Electron Microscopy (Fig. 1g).

### *Mlkl*^*D139V*^ causes a lethal perinatal inflammatory syndrome

**Fig. 2:**
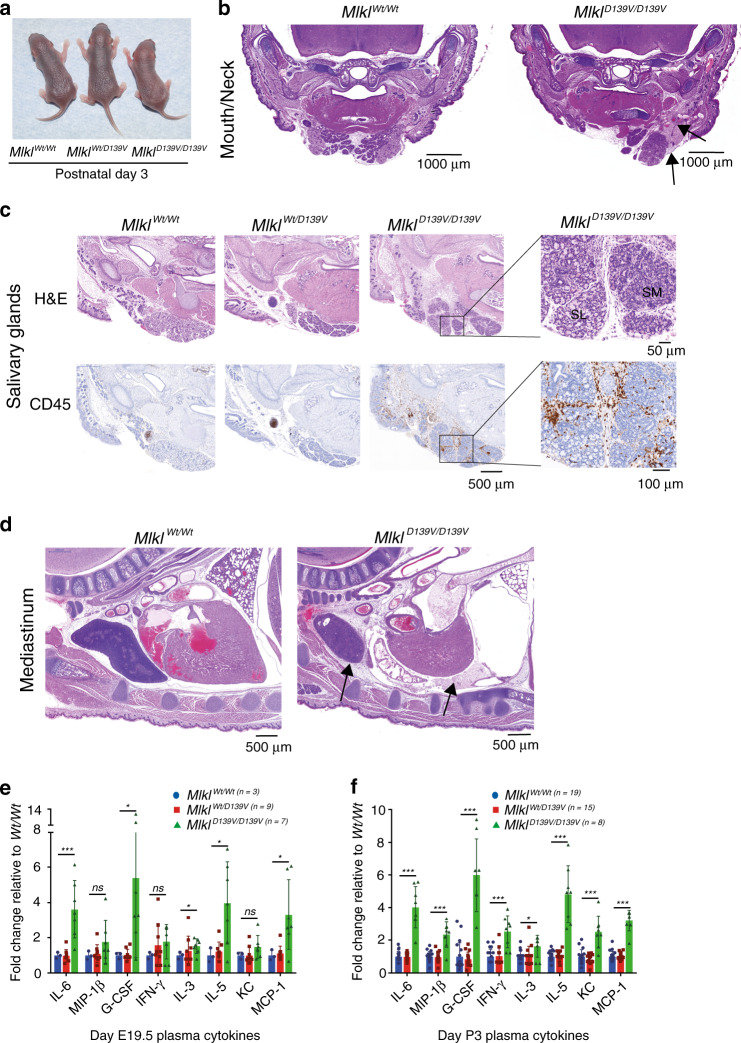
Homozygous *Mlkl*^*D139V*^ neonates exhibit dispersed upper body inflammation. **a** Macroscopic appearance of *Mlkl*^*Wt/Wt*^, *Mlkl*^*Wt/D139V*^ and *Mlkl*^*D139V/D139V*^ mice at postnatal day 3. **b** Coronal section of mouth and neck region of postnatal day 2 litter mates stained with hematoxylin and eosin (H&E). Dilated blood vessels and edema are indicated by arrows. **c** Serial mandible sections from postnatal day 3 litter mates stained with H&E and anti-CD45. Inset black boxes are magnified in right panel. SL, sublingual gland. SM, submandibular gland. Images representative of n = 3–4 P3 pups per genotype. **d** H&E stained sections from mediastinum of postnatal day 2 litter mates. Thymic cortical thinning and pericardial infiltration are indicated by arrows. For full anatomical annotations for (**b**) and (**d**) see Supplementary Fig. 2h. (**b**) and (**d**) representative of *n* = 5–6 P2 pups examined with similar characteristics. Scale bars for (**b**–**d**) range from 50 to 1000 μm as indicated. Multiplex measurement of plasma cytokine levels at E19.5 (**e**) and postnatal day 3 (**f**). Each symbol represents one independent pup sampled; *Mlkl*^*Wt/W*t^ – blue circles, *Mlkl*^*Wt/D139V*^- red squares, *Mlkl*^*D139V/D139V*^- green triangles, with bar height and error bars representing mean ± SD respectively for *n* = 3–to 19 pups as indicated. *p ≤ 0.05, **p ≤ 0.01, ***p ≤ 0.005 calculated using an unpaired, two-tailed *t*-test.

To define the phenotypic consequences of constitutively active MLKL in the absence of any confounding effects resulting from *Mpl*-deficiency, all subsequent studies were performed on a *Mpl*^*+/+*^ background. Homozygous *Mlkl*^*D139V/D139V*^ pups were born at expected Mendelian frequencies (Supplementary Table 1) and were ostensibly normal macroscopically and histologically at E19.5 (Supplementary Fig. 2a–d). However, by 3 days of age, although outwardly indistinguishable from littermates (Fig. 2a), they exhibited reduced body weight (Supplementary Fig. 2b) and failed to thrive, with a maximum observed lifespan of 6 days under conventional clean housing conditions. Like *Mlkl*^*Wt/D139V*^ mice, *Mlkl*^*null/D139V*^ compound heterozygotes were present at the expected frequency at P21 and developed normally to adulthood (Supplementary Table 2). Thus, the constitutive activity of MLKL^D139V^ was not affected by the presence of normal MLKL protein suggesting it is the absolute allelic dose of *Mlkl*^*D139V*^ that determines perinatal lethality. To confirm that the phenotype of the ENU derived *Mlkl*^*D139V*^ mice was due to the *Mlkl*^*D139V*^ missense mutation, we independently generated *Mlkl*^*D139V*^ mice using CRISPR-Cas9 genomic editing. Homozygote CRISPR-*Mlkl*^*D139V/D139V*^ mice also died soon after birth (Supplementary Table 3).

Hematoxylin-Eosin stained-sections from both P2 and P3 *Mlkl*^*D139V/D139V*^ pups revealed multifocal acute inflammation characterized by neutrophilic infiltration, dilated blood vessels and edema (Fig. 2b) in the dermis and subcutis of the head and neck. These inflammatory features were not observed in *Mlkl*^*Wt/Wt*^ or *Mlkl*^*Wt/D139V*^ littermates, nor in *Mlkl*^−/−^ mice of the same age (Supplementary Fig. 2i). Cells of hematopoietic origin, revealed by immunohistochemical staining for CD45, were sparsely distributed throughout the lower head and neck and confined predominantly to a clearly delineated developing lymph node in *Mlkl*^*Wt/Wt*^ and *Mlkl*^*Wt/D139V*^ littermates (Fig. 2c). In contrast, CD45^+^ cells were more numerous and distributed throughout the cutis, subcutis and salivary glands of *Mlkl*^*D139V/D139V*^ pups (Fig. 2c). A mixture of diffuse and focal inflammatory infiltration was also observed within the mediastinum and pericardial space of all P2/P3 *Mlkl*^*D139V/D139V*^ pups examined, as was a paucity of thymic cortical lymphocytes (Fig. 2d, Supplementary Fig. 2e), phenotypes not evident in E19.5 embryos (Supplementary Fig. 2d). No other consistent lesions were observed by histopathology. Consistent with this inflammatory phenotype, significantly elevated levels of several pro-inflammatory cytokines and chemokines were evident in the plasma of both E19.5 and P3 *Mlkl*^*D139V/D139V*^ pups (Fig. 2e, f). Blood glucose levels were normal (Supplementary Fig. 2f, g).

### Hematopoietic defects in *Mlkl*^*D139V*^ mice

**Fig. 3:**
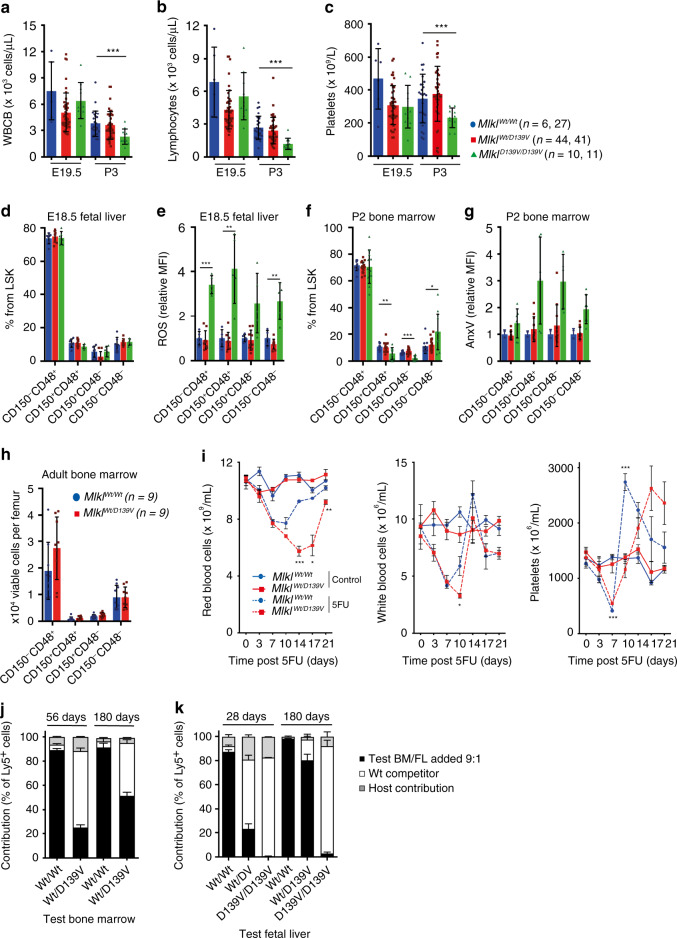
Alterations in hematopoietic cells and defective emergency hematopoiesis in *Mlkl*^D139V^ mice. **a**–**c** Absolute white blood cell (WBCB), lymphocyte and platelet numbers in peripheral blood of E19.5 and P3 pups, *n* = 6, 27, 44, 41, 10, and 11 as indicated. **d** Proportions of HSC (Lineage^-^Sca-1^+^c-kit^+^ (LSK) CD150^+^ CD48^−^), MPP (LSK CD150^−^ CD48^−^), HPC-1 (LSK CD150^−^ CD48^+^) and HPC-2 (LSK CD150^+^ CD48^+^)^82^, *n* = 5 per genotype and (**e**) relative levels of ROS (*n* = 4, 9, 5) (**f**) P2 bone marrow LSK populations (*n* = 9, 18, and 11) and (**g**) relative AnnexinV binding (*n* = 2, 11, 7). (**h**) HSC subtypes in adult bone marrow, *n* = 9 per genotype. **a**–**h** Each symbol represents one independent animal; *Mlkl*^*Wt/W*t^ – blue circles, *Mlkl*^*Wt/D139V*^- red squares, Mlkl^D139V/D139V^- green triangles, with bar height and error bars representing mean ± SD respectively, or range when *n* = 2. Red and white blood cells and platelets in *Mlkl*^*Wt/Wt*^ (blue circles) and *Mlkl*^*Wt/D139V*^ (red squares) mice after treatment with 150 mg per kg 5FU or saline. Means ± SEM from one experiment in which three mice were sampled at each time point for each treatment group, similar results were obtained in an independent cohort. **j** Bone marrow from *Mlkl*^*Wt/Wt*^ or *Mlkl*^*Wt/D139V*^ mice on CD45^Ly5.2^ background was mixed with wild-type CD45^Ly5.1^ competitor bone marrow and transplanted into irradiated CD45^Ly5.1/Ly5.2^ recipients. Peripheral blood mononuclear cells (PBMCs) quantified after 56 and 180 days. Mean ± SEM are shown (3 donors per genotype, 3–5 recipients per donor). **k** Fetal liver cells (CD45^Ly5.2^; *Mlkl*^*Wt/Wt*^, *Mlkl*^*Wt/D139V*^ or *Mlk*^*D139V/D139V*^) were transplanted into lethally irradiated recipients (CD45^Ly5.1/Ly5.2^) together with competitor bone marrow (CD45^Ly5.1^). Contribution to PBMCs 28 days and 180 days after transplantation. Mean ± SEM are shown (2–10 donors per genotype, 2–6 recipients per donor). Host contribution (CD45^Ly5.1/Ly5.2^) is depicted in gray, competitor (CD45^Ly5.1^) in white, and test (CD45^Ly5.2^) in black for (**j**) and (**k**). *p ≤ 0.05, **p ≤ 0.01, ***p ≤ 0.005 calculated using an unpaired, two-tailed *t*-test.

Although blood cell numbers were unchanged in *Mlkl*^*D139V/D139V*^ pups at E19.5 relative to *Mlkl*^*Wt/Wt*^ and *Mlkl*^*Wt/D139V*^ littermates, by P3 significant deficits were evident in total white blood cell count (due predominantly to reductions in lymphocyte numbers) and platelet numbers (Fig. 3a–c, Supplementary Fig. 3a). Similarly, the numbers of hematopoietic stem and progenitor cells were present at normal proportions in fetal livers of E18.5 *Mlkl*^*D139V/D139V*^ pups, although increased levels of intracellular ROS were uniformly evident in live cells, (Fig. 3d, e, Supplementary Fig. 3b,c). By P2, deficits in CD150^+^CD48^+^ and CD150^+^CD48^−^ populations were present (Fig. 3f), accompanied by increased AnnexinV binding in live cells (which indicates phosphatidyl serine exposure) of all lineages (Fig. 3g). In adult *Mlkl*^*Wt/D139V*^ mice, numbers of hematopoietic stem and progenitor cells were unaffected (Fig. 3h); however, upon myelosuppressive irradiation, recovery of hematopoietic cell numbers was delayed and characterized by increased expression of ROS and Annexin V (Supplementary Fig. 3d, e). When challenged with the cytotoxic drug 5-fluorouracil (5-FU), blood cell recovery in *Mlkl*^*Wt/D139V*^ mice was similarly delayed (Fig. 3i). In competitive transplants in which test *Mlkl*^*Wt/D139V*^ or *Mlkl*^*Wt/Wt*^ marrow was co-injected with wild-type competitor marrow in 10:1 excess, as expected, *Mlkl*^*Wt/Wt*^ marrow contributed to 90% of recipient blood cells 8 weeks after transplantation and maintained that level of contribution for 6 months (Fig. 3j). In contrast, *Mlkl*^*Wt/D139V*^ marrow performed poorly, contributing to 25% and 51% of recipient blood cells at these times (Fig. 3j). Similarly, while wild-type fetal liver cells contributed to the vast majority of blood cells in irradiated recipients up to 6 months after transplantation, cells from *Mlkl*^*D139V/D139V*^ embryos failed to compete effectively during this period (Fig. 3k). Heterozygote *Mlkl*^*Wt/D139V*^ fetal liver cells contributed poorly in the first month following the graft but recovered to contribute more after six months (Fig. 3k). Thus, while tolerated under steady-state conditions, heterozygosity of *Mlkl*^*D139V*^ is deleterious under conditions of hematopoietic stress. Bone marrow- derived HSCs from *Mlkl*^*Wt/D139V*^ adults and fetal liver- derived HSCs from *Mlkl*^*Wt/D139V and*^ *Mlkl*^*D139V/D139V*^ pups also formed fewer and smaller colonies in the spleens of lethally irradiated recipient mice after 8 days (Supplementary Fig. 3f).

### *Mlkl*^*D139V*^ fibroblasts are less sensitive to necroptosis

**Fig. 4:**
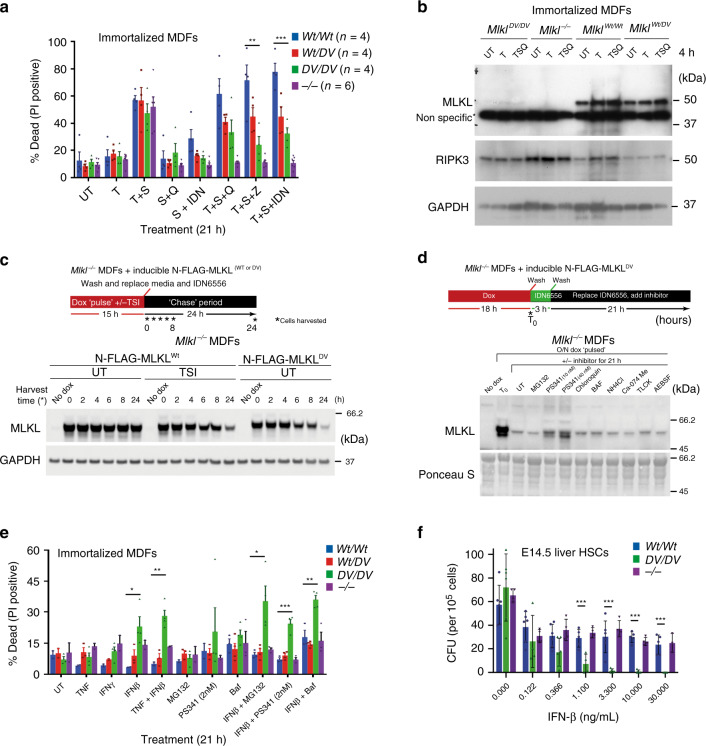
MLKL^D139V^ undergoes constitutive post-translation turn-over. MDFs were isolated from *Mlkl*^*Wt/Wt*^, *Mlkl*^*Wt/D139V*^, *Mlk*^*D139V/D139V*^ or *Mlkl*^−/−^ *pups*, immortalized and stimulated as indicated for 21 h for quantification of PI-positive cells using flow cytometry (*n* = 4, 4, 4, and 6) (**a**), or for 4 h for western blot analysis (**b**). *Mlkl*^−/−^ MDFs were stably transduced with doxycycline-inducible FLAG-MLKL^WT^ and FLAG-MLKL^D139V^ constructs to examine MLKL protein stability after doxycycline withdrawal (**c**) and in the presence of indicated compounds (FLAG-MLKL^D139V^) (**d**). **e** Immortalized MDFs from (**a**) were stimulated as indicated for 21 h for quantification of PI-positive cells using flow cytometry (*n* = 2–3, 3–4, 4, 2–3). **f** E14.5 fetal liver cells from *Mlkl*^*Wt/Wt*^, *Mlk*^*D139V/D139V*^ or *Mlkl*^−/−^ embryos were plated in the presence of indicated dose of IFN-β and colonies enumerated after 7 days (*n* = 4–6). (**a**, **e** and **f**) represent mean ± SEM (A,E) or ±SD (**f**). **b**–**e** Representative images of at least three similar experiments. *p ≤ 0.05, **p ≤ 0.01, ***p ≤ 0.005 calculated using an unpaired, two-tailed *t*-test.

To examine if the constitutive activity of exogenously expressed MLKL^D139V^ results in an enhanced propensity for necroptosis in cells that express MLKL^D139V^ under the control of its endogenous promoter, we immortalized MDFs from *Mlkl*^*Wt/Wt*^, *Mlkl*^*Wt/D139V*^ and *Mlkl*^*D139V/D139V*^ littermates and from *Mlkl*^−/−^ E19.5 pups. We observed no significant differences in basal cell death levels, nor any differences in the sensitivity of these cells to an apoptotic stimulus such as TNF plus Smac mimetic (Fig. 4a, Supplementary Fig. 4a). Surprisingly and in apparent contradiction to our initial observations using exogenous expression systems, endogenous expression of this *Mlkl* mutant revealed a significant and consistent decrease in sensitivity to TNF-induced necroptosis using three different pan-caspase inhibitors Q-VD-OPh, zVAD-fmk and IDN-6556/emricasan in a *Mlkl*^*D139V*^ dose-dependent manner (Fig. 4a, Supplementary Fig. 4a). MDFs isolated from *Mlkl*^*D139V/D139V*^ homozygotes were up to 60% less sensitive to TNF-induced necroptosis compared with *Mlkl*^*Wt/Wt*^ MDFs, but were not as resistant as *Mlkl*^−/−^ MDFs (Fig. 4a).

While there were no obvious differences in the levels of MLKL^Wt^ and MLKL^D139V^ protein following doxycycline induced exogenous expression (Fig. 1f), MLKL was virtually undetectable by Western blot in *Mlkl*^*D139V/D139V*^ pup-derived fibroblasts immortalized and cultured ex vivo (Fig. 4b). There was, however, no significant reduction in *Mlkl* gene transcript levels in these cells (Supplementary Fig. 4b) suggesting that this reduction was post-transcriptional. A reduction in MLKL^D139V^ protein levels was also evident in whole E14.5 embryo protein lysates and in single cell clones derived from HOXA9 factor dependent myeloid cell lines derived from *Mlkl*^*D139V/D139V*^ E14.5 embryos (Supplementary Figs. 4c, j). Lysates from E14.5 embryos also clearly show that *Mlkl*^*Wt/D139V*^ heterozygotes have intermediate levels of MLKL, reflecting the intermediate sensitivity of *Mlkl*^*Wt/D139V*^ MDFs to necroptotic stimuli (Supplementary Fig. 4c and Fig. 4a).

### MLKL^D139V^ protein turnover requires proteasome activity

Measuring the half-life of endogenously expressed MLKL^D139V^ is not possible using conventional ‘pulse chase’ methods because this mutant protein induces necroptotic cell death, so we capitalized on our previous observation that an N-terminally FLAG-tagged MLKL 4HB forms a high molecular weight membrane-associated complex just like the untagged form, but, unlike the untagged version, does not kill cells^9^. Consistent with this observation, N-FLAG full-length mouse MLKL^D139V^ did not induce cell death when inducibly expressed in *Mlkl*^*−/−*^ MDFs (Supplementary Fig. 4f).

Using this system, we were able to measure the cellular turn over of MLKL by inducing N-FLAG-MLKL^WT^ or N-FLAG-MLKL^D139V^ expression in *Mlkl*^−/−^ MDFs for 15 h using doxycycline then washing and culturing them in the absence of doxycycline for a further 2–24 h. In the absence of a stimulus (UT), the levels of N-FLAG-MLKL^WT^ remained consistent over the 24-h period (Fig. 4c), indicating that non-activated wild-type MLKL is a stable protein in MDFs. However, when these cells were treated with a necroptotic stimulus (TSI) to activate MLKL, the levels of wild-type MLKL rapidly declined even though these cells were unable to undergo a necroptotic cell death. Consistent with the fact that untagged MLKL^D139V^ behaves as an auto-activated form of MLKL (Fig. 1e), the half-life of N-FLAG-MLKL^D139V^ (4–6 h) was similar to the WT version stimulated with TSI (Fig. 4c). Thus, the absence of endogenously expressed MLKL^D139V^ in E14.5 embryo lysates and cultured fibroblasts can be attributed to the reduced post-translational stability of this mutant auto-activated form of the protein.

To determine which cellular mechanism(s) are required for the clearance of activated MLKL^D139V^, we included a series of proteasome, lysosome and specific protease inhibitors during the ‘chase’ period after doxycycline was withdrawn (schematic in Fig. 4d). The doses of all inhibitors were carefully titrated and combined with pan-caspase inhibitor IDN6556 to minimize any toxicity-associated apoptotic cell loss during the chase period. To exclude any confounding RIPK3-mediated activation of the necroptotic pathway by proteasome inhibitors^41^ (Supplementary Fig. 4f), the same experiment was also performed in *Mlkl*^−/−^, *RIPK3*^−/−^ MDFs (Supplementary Fig. 4d). Even at the very low doses used, addition of the proteasome inhibitor PS341 was accompanied by reduced clearance of N-FLAG-MLKL^D139V^ and the stabilization of higher molecular weight species that resemble mono- and poly-ubiquitinated MLKL (Fig. 4d, Supplementary Fig. 4d, i). This PS341 mediated protection of activated MLKL was also evident when the same assay was performed for phospho(p)S345-N-FLAG-MLKL^WT^ (Supplementary Fig. 4e). The less potent proteasome inhibitor MG132 did not stabilize MLKL^D139V^ to levels that could be resolved by western blotting of total MLKL in this assay but did facilitate some stabilization of (p)-N-FLAG-MLKL^WT^. Chloroquine, Bafilomycin and NH_4_Cl also partially protected against (p)-N-FLAG-MLKL^WT^ clearance, supporting the potential role for lysosome mediated degradation of natively phosphorylated MLKL^WT^^15,17^, but this was not observed for constitutively activated N-FLAG-MLKL^D139V^ using this approach (Fig. 4d, Supplementary Fig. 4d).

Based on these findings we hypothesized that this MLKL-clearance mechanism limits the capacity of MLKL^D139V^ to kill *Mlkl*^*Wt/D139V*^ and *Mlkl*^*D139V/D139V*^ cells in culture and in vivo by maintaining protein levels below a critical threshold. To test whether this protective mechanism could be overwhelmed, we incubated MDFs with agents that have been shown to induce *Mlkl* expression (TNF, interferons (IFN) β and γ)^42–44^, or inhibit its turnover (proteasome and lysosome inhibitors). MLKL^D139V^ protein in untreated *Mlkl*^*D139V/D139V*^ MDFs was undetectable by Western blot but became faintly detectable following addition of these stimuli (Fig. 4b and Supplementary Fig. 4g). This correlates with moderate but statistically significant increases in cell death (particularly when compared with the lack of sensitivity to conventional necroptotic stimuli (Fig. 4a)), when exposed to IFN-β alone and in combination with proteasome or lysosome inhibitors (Fig. 4e). A similar allele-dose dependent sensitivity is also evident in primary MDFs (Supplementary Fig. 4h). To examine if this mechanism may explain the reduced capacity of *Mlkl*^*D139V/D139V*^ fetal liver cells to reconstitute an irradiated host (Fig. 2k), ex vivo colony forming assays were performed on fetal liver cells derived from *Mlkl*^*Wt/Wt*^ and *Mlkl*^*D139V/D139V*^ E14.5 littermates, alongside E14.5 livers taken from *Mlkl*^−/−^ mice. *Mlkl*^*D139V/D139V*^ cells showed significantly increased sensitivity to the inhibitory effects of IFN-β, with reduced colony formation at low doses of cytokine that affected *Mlkl*^*Wt/Wt*^ and *Mlkl*^−/−^ colony formation only marginally (Fig. 4f). Factor dependent myeloid cells generated through HOXA9 immortalization of E14.5 liver HSCs also demonstrated high rates of cell death under conventional FDM culture conditions when derived from *Mlkl*^*Wt/D139V*^ or *Mlkl*^*D139V/D139V*^ embryos (Supplementary Fig. 4k). Together, these experiments provide further evidence for the existence of steady-state MLKL surveillance and turnover mechanisms that suppress cell death by lowering the abundance of activated MLKL below a killer threshold at the cellular level^6,14–16^ and provide an in vivo precedent for both the existence of this phenomenon and the lethal consequences of its dysregulation in the form of the *Mlkl*^*D139V*^ mouse.

To test whether the lethal inflammation in *Mlkl*^*D139V/D139V*^ neonates was mediated by the direct or indirect activation of the inflammasome by active MLKL we crossed this line with the *Caspase 1/11* null mouse strain^45–47^. This did not enhance the lifespan of *Mlkl*^*D139V/D139V*^ pups (Table 1). The combined genetic deletion of *Casp8* and *Ripk3* did not rescue or extend the life of *Mlkl*^*D139V/D139V*^ mice, indicating that postnatal lethality is not mediated by bystander extrinsic apoptotic cell death that may occur secondary to initial waves of MLKL^D139V^-mediated necroptosis (Table 1). The genetic deletion of *Tnfr1, Myd88* or *Ifnar* individually did not provide any extension to the lifespan of *Mlkl*^*D139V*^ homozygote pups (Table 1). These data indicate that the removal of any one of these routes to NF-κB- and interferon-mediated gene upregulation, inflammation or apoptotic cell death is not sufficient to protect mouse pups against a double allelic dose of *Mlkl*^*D139V*^.

**Table 1:** Postnatal lethality in *Mlkl*^*D139V/D139V*^ homozygotes is independent of *Tnfr1, Myd88, Ripk3*, *Casp8, Casp1,* and *Casp11*.

Stage genotyped	E14	E18	P21	P21	P21	P21	P21	P21	P21
C57BL/6 genetic background	*Wt*	*Wt*	*Wt*	*Tnfr1*^*−/−*^	*Myd88*^−/−^	*Ripk3*^*−/−*^, *C8*^+/−^	*Ripk3*^−/−^, *C8*^−/−^	*Ifnar*^−/−^	*C1*^−/−^*, C11*^−/−^
*Mlkl*^*Wt/Wt*^	58 (39)	7 (9)	15 (11)	19 (15)	3 (2)	10 (6)	2 (2)	15 (11)	10 (8)
*Mlkl*^*Wt/D139V*^	70 (78)	17 (18)	30 (22)	41 (30)	6 (4)	14 (12)	5 (4)	30 (22)	21 (16)
*Mlkl*^*D139V/D139V*^	28 (39)	13 (9)	0 (11)	0 (15)	0 (2)	0 (6)	0 (2)	0 (11)	0 (8)
Total # genotyped	156	37	45	60	9	24	7	45	31

() - number of pups expected from Mendelian segregation, calculated from total number of pups genotyped and rounded to the nearest whole number. Gene names italicized.

E, embryonic day; P, days postnatal.

### Common human missense *MLKL* variants map to the brace region

**Fig. 5:**
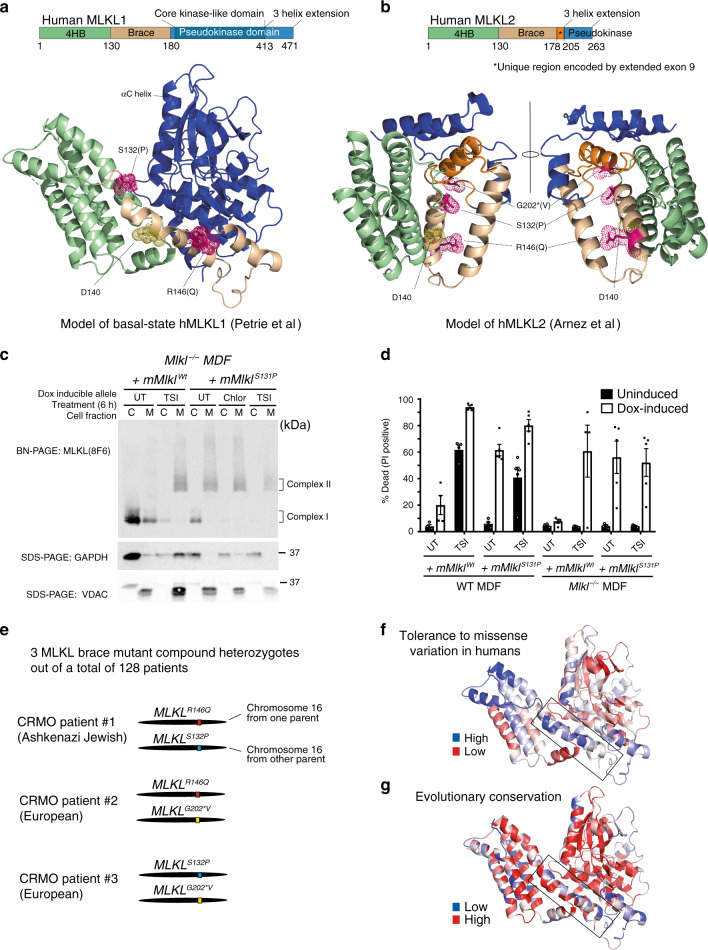
Three of the four highest frequency missense human *MLKL* SNPs encode non-conservative amino acid substitutions within or adjacent to the brace helix region. **a** S132 and R146 (magenta) are located on either side of D140 (yellow—equivalent to mouse D139) in the first human MLKL brace helix. Alternate amino acids encoded by human polymorphisms indicated in parentheses. **b** G202 is predicted to be on an α helix unique to MLKL2 isoform and to form an interface along with S132 and R146. The mouse equivalent of human rs35589326 (hMLKL^S132P^), mMLKL^S131P^, spontaneously forms membrane-associated high molecular weight complexes following Blue Native (BN) PAGE (**c**) and kills MDFs (**d**) in the absence of extrinsic necroptotic stimuli when expressed in mouse dermal fibroblasts for 6 (**c**) and 21 hrs respectively (**d**). C; cytoplasmic fraction, M; crude membrane fraction, TSI; TNF, Smac-mimetic and IDN6556, Chlor: Chloroquine. **c** Representative of two independent experiments with similar results. Error bars in (**d**) indicate the mean ± SEM of 4–5 independent experiments. **e** Schematic showing brace helix variant combinations identified as alleles in trans in three CRMO patients. **f** MTRs are mapped onto the structure of MLKL to show regions that have low tolerance to missense variation in the human population (red) and regions that have increased tolerance to missense variation (blue), normalized to the gene’s MTR distribution. **g** Multiple sequence alignment (MSA) conservation scores are mapped onto the structure of MLKL to show regions that are highly conserved through evolution (red) and regions that are less conserved through evolution (blue).

Given the severe inflammatory phenotype of murine *Mlkl*^*D139V/D139V*^ neonates and the significant defects in stress hematopoiesis observed in murine *Mlkl*^*Wt/D139V*^ adults, we explored the prevalence of brace region variation in human *MLKL*. Examination of the gnomAD database^48^, which contains human *MLKL* exome or genome sequence data from a total of over 140,000 individuals revealed that the second and third highest frequency human *MLKL* missense coding variants; rs34515646 (R146Q) and rs35589326 (S132P), alter the same brace helix (Table 2, Fig. 5a). The 4th most common human *MLKL* polymorphism, rs144526386 (G202*V) is a missense polymorphism identified exclusively in the context of a shorter splice isoform of MLKL (*) named ‘MLKL2’^49^ (Table 2, Fig. 5b). The full-length canonical transcript of *MLKL* encodes a 471 amino acid protein, while MLKL2 is an alternatively spliced isoform of MLKL that is 263 amino acids long. MLKL2 lacks a large portion of the pseudokinase domain which functions to repress the killing potential of the 4HB domain^6–9^ and recruit co-effectors like RIPK3 and HSP90^13,50–52^. Glycine202* is encoded by an extension to exon 9 that is unique to the *MLKL2* splice isoform (Fig. 5a, b).

**Table 2:** Human *MLKL* brace helix polymorphism frequency human *MLKL* SNP.

Feature	R146Q – rs34515646	S132P – rs35589326	G202^a^V – rs144526386
CADD Score (phred-scaled)	0.407	6.381	3.825
UK Biobank – Total MAF (*n*)	0.0253 (487,658)	0.0161 (487,625)	0.0147 (487,488)
gnomAD – Total MAF (*n*)	0.0152 (141,339)	0.0138 (141,442)	0.01228 (141,400)
gnomAD – Highest MAF (*n*) population	0.0252 (64,541) European (Non-Finnish)	0.0311 (5185) Ashkenazi Jewish	0.0245 (5184) Ashkenazi Jewish
1000 genomes – Total MAF (*n*)	0.0052 (2504)	0.0088 (2504)	0.0102 (2504)
1000 genomes – Highest MAF (*n*) population	0.018 (503) European	0.024 (489) South Asian	0.021 (503) European

Gene names italicized.

*N* number of individuals sequenced, *MAF* Minor Allele Frequency – count.

^a^Alternative transcript.

While the amino acid substitution *MLKL*^*R146Q*^ is classified as ‘tolerated’ and ‘benign’ by SIFT/POLYPHEN 2 algorithms^53,54^ (Supplementary Table 1), R146 of human MLKL shows NMR chemical shift perturbations in the presence of the negatively charged IP3 and IP6 phospholipid head groups, indicating a possible role in membrane association and disruption^11,55^. Ser-132 lies before the first structured residue of the first brace helix in human MLKL (Fig. 5a)^13,56,57^. A Serine-to-Proline substitution at this position is predicted to significantly impact the conformation of the immediately adjacent W133 (brace helix) and in turn, the proximal W109 within the 4HB domain (Supplementary Fig. 5a). When mapped to a model of MLKL splice-isoform 2^49^ Glycine 202* is predicted to be on an isoform 2-specific helix and to form an interface along with S132 and R146 of brace helix 1. While the precise structural consequence of these three brace polymorphisms is unknown, modeling of human MLKL predicts that disruption in the brace region favors adoption of an activated conformation^13^. Consistent with this prediction, the murine equivalent of the human S132P variant, mMLKL^S131P^, formed high molecular weight membrane-associated complexes and killed MDFs in the absence of a necroptotic stimulus (Fig. 5c, d) when expressed at close to endogenous levels (Supplementary Fig. 5b). Similarly to mMLKL^D139V^, unstimulated mouse dermal fibroblasts generated from the first generation of heterozygote and homozygote mutant pups of a recently generated *mMlkl*^*S131P*^ CRISPR modified mouse line demonstrated a clear reduction in MLKL protein levels relative to those prepared from wild-type littermates (Supplementary Fig. 5c), though the cellular clearance is not as complete as observed for mMLKL^D139V^. Together, these data indicate that constitutive activation and reduced protein stability is not a unique, idiosyncratic feature of the mMLKL^D139V^, but also a feature of a closely situated MLKL brace mutant, mMLKL^S131P^.

### MLKL brace variants occur in trans more frequently in CRMO

To investigate if human MLKL brace region polymorphisms play a role in human autoinflammatory disease we examined their frequency in cohorts suffering from ankylosing spondylitis (AS), chronic recurrent multifocal osteomyelitis (CRMO), Guillain Barré Syndrome (GBS) and Synovitis, Acne, Pustulosis, Hyperostosis and Osteitis (SAPHO) Syndrome. The individual minor allele frequencies of R146Q, S132P, and G*202V are not enriched in these disease cohorts relative to healthy controls when population distribution is accounted for (Supplementary Tables 4 and 5). However, these alleles occur in trans (making ‘compound heterozygotes’—schematic in Fig. 5e) in 3 out of 128 CRMO patients. This is 29 times the frequency that these combinations are observed in healthy NIH 1000 genomes samples (where there are only two compound heterozygotes for these polymorphisms out of 2504 healthy individuals sequenced), or at 10–12 times the frequency when only European CRMO patients and two separate healthy European control populations were compared (Table 3).

**Table 3:** Human *MLKL* brace helix compound heterozygotes in CRMO vs healthy controls.

Population	Frequency of relevant compound Hets^a^ in CRMO	Frequency of relevant compound Hets^a^ in healthy controls	CRMO:Healthy^b^	2 tailed *P* value (Fisher’s exact)	2 tailed *P* value (chi square with Yate’s)
Global^c^	0.023 (3/128)	0.0008 (2/2504) NIH 1KG	29:1	0.001	0.0001
European^d^	0.02 (2/101)	0.002 (1/503) NIH 1KG	10:1	0.074	0.1215
European^d^	0.02 (2/101)	0.0017 (25/14,542) QUT controls	12:1	n/a	0.0022

^a^Combinations of R146Q – rs34515646, S132P – rs35589326 and G202*V – rs144526386 (Fig. 5e).

^b^Frequency ratio rounded to nearest whole number.

^c^CRMO patients and healthy controls of all ancestries included.

^d^CRMO patients and healthy controls of only European descent included.

## Discussion

In contrast to apoptosis, necroptosis is widely considered to be an inflammatory form of cell death. However, definitive evidence for this proposition has yet to emerge. Because MLKL is activated by inflammatory stimuli such as TNF it is very difficult to separate cause from effect. The serendipitous identification of an auto-activating mutant of MLKL (*Mlkl*^*D139V*^) in mice has allowed us to explore the consequences of inappropriate necroptosis in the absence of such confounding factors. Furthermore, it has led to significant insights into the critical adult hematopoietic and perinatal developmental processes that are most sensitive to excessive MLKL activation, and into physiological mechanisms that have evolved to neutralize activated MLKL.

In the absence of a robust immunohistochemical marker for RIPK3-independent necroptosis, it is not possible to pinpoint exactly which cell type/s undergo necroptosis in *Mlkl*^*D139V*^ mice. Nevertheless, the presence of high levels of circulating pro-inflammatory cytokines in *Mlkl*^*D139V/D139V*^ pups at E19.5 relative to *Mlkl*^*Wt/Wt*^ and *Mlkl*^*Wt/D139V*^ littermates suggests that necroptosis and ensuing inflammation begins in the sterile in utero environment. This is not enough to overtly retard prenatal development or affect hematopoietic cell populations. However, upon birth and/or exposure to the outside environment the capacity of homozygous *Mlkl*^*D139V/D139V*^ pups to suppress *MLKL*^*D139V*^ activity is overwhelmed and they die within days of birth. This is clearly a dose-dependent effect because both *Mlkl*^*D139V/Wt*^ and *Mlkl*^*D139V/null*^ heterozygous mice are viable. Postnatal death cannot be prevented by combined deficiencies in *Ripk3* and *Casp8* nor by deficiency of other important inflammatory genes including *Tnfr1, Myd88* or *Ifnar*. In light of the elevated levels of circulating G-CSF, IL-6 and IL-5 observed, the role of these key mediators in the initiation or potentiation of pre- and perinatal inflammation in *Mlkl*^*D139V/D139V*^ pups will be the subject of future investigations.

The *Mlkl*^*D139V*^ mutation was initially identified for its capacity to moderately increase platelet production independent of the thrombopoietin receptor Mpl. While the mechanism underlying this observation remains unclear, it follows observations by others that another member of the necroptotic pathway, RIPK3, plays a role in platelet activation^58^. The reduced platelet levels observed in *Mlkl*^*D139V/D139V*^ pups is unlikely to be the sole cause of death given much more severe thrombocytopenia is not lethal in *Mpl*^−/−^ mice^40^. Difficulty with suckling due to inflammatory infiltration of the head and neck and resulting failure to thrive is one possible explanation for the lethality in *Mlkl*^*D139V/D139V*^ pups. However, the narrow window of mortality for these pups and marked pericardial immune infiltration make heart failure another potential cause of sudden neonatal death.

The *Mlkl*^*D139V*^ mouse reveals that maintaining MLKL levels below a threshold can prevent necroptotic activation. This strain is a potential tool for the mechanistic and physiological examination of MLKL-mediated extracellular vesicle generation or other cell death-independent roles related to inflammation that is unconfounded by RIPK3 activation. While others have recently shown that an ESCRT dependent repair or extracellular vesicle extrusion can help protect membranes from limited MLKL damage^14–16^, and that p-MLKL can be internalized and degraded by the lysosome^17^ our data also suggest a role for the proteasome in the disposal of activated MLKL, be it directly, or in its capacity to generate free ubiquitin. This creates the possibility that these mechanisms or the previously described ESCRT mechanisms intersect in some way. Finally, the ability of these mechanisms to hold single gene-dose levels of active MLKL in check without deleterious consequences in vivo supports the idea that direct inhibition of activated MLKL may be an effective means to therapeutically prevent unwanted necroptotic cell death. Similarly, the *Mlkl*^*D139V*^ mouse and assorted relevant crosses may prove to be a useful tool for the further examination of whether ROS production is co-incident with-, causative of- or consequential to- necroptotic plasma membrane disruption in varied tissue types and under highly physiologically relevant contexts (recently reviewed by^59^).

While any mouse MLKL-human MLKL comparisons must be made cautiously in light of the species-specific structural and mechanistic differences^5,12,13^, it is notable that out of over 140,000 individuals surveyed, there is only one recorded case of a human carrying a substitution equivalent to the *mMlkl*^*D139V*^ mouse (*hMLKL*^*D140V*^; rs747627247) in the gnomAD database, and this individual is heterozygous for this variant. To our surprise, 3,841 individuals in gnomAD (55 of which are homozygotes) carry a very closely situated MLKL brace region variant –*MLKL*^*S132P*^. Our CRISPR-generated *Mlkl*^*S131P*^ mouse equivalent supports the connection between constitutive MLKL activation and decreased MLKL protein stability. Preliminary observations show that this variant manifests in a much milder and context specific phenotype in mice than *mMlkl*^*D139V*^, which is consistent with its high frequency presence in the human population.

Overlaid with structural, biochemical, cell and animal-based evidence of function, it is tempting to speculate that these human MLKL brace region variants lead to altered MLKL function and/or regulation in what is most likely a highly tissue-, context- or even pathogen specific way^60–62^. While increased numbers and examination of independent cohorts will be required to confirm the statistical enrichment of human MLKL brace variants occurring in *trans* in the autoinflammatory disease CRMO, this patient cohort offers a tantalizing clue into their potential as modifiers of complex, polygenic inflammatory disease in present day humans.

## Methods

### Mice

All mice were backcrossed to C57BL/6 mice for >10 generations or generated on a C57BL/6J background. *Mlkl*^−/−^, *Tnfr1*^−/−^*, Myd88*^*−/−*^*, IFNAR1*^−/−^*, Ripk3*^−/−^*, Casp8*^−/−^, and *Casp1/Casp11*^−/−^ *mice* were generated as described^4,45,46,63–67^. Mice designated as E19.5 were obtained by Caesarean section from mothers that received progesterone injections at E17.5 and E18.5. Independent mouse strains that carry the D139V or S131P mutation in the *Mlkl* gene (MLKL^D139V^ CRISPR) were generated using CRISPR/Cas9 as previously described^68^. For D139V, one sgRNA of the sequence GGAAGATCGACAGGATGCAG (10 ng per μL), an oligo donor of the sequence ATTGGAATACCGTTTCAGATGTCAGCCAGCCAGCATCCTGGCAGCAGGAAGATCGACAGGTTGCAGAAGAAGACGGgtgagtctcccaaagactgggaaagagtaggccagggttgggggtagggtgg (10 ng per μL) and Cas9 mRNA (5 ng per μL) were injected into the cytosol of C57BL/6J zygotes. Mice were sequenced across the mutated region to confirm incorporation of the altered codon and analysis was performed after at least 2 back-crosses to C57BL/6. The same procedure was followed for the generation of MLKL^S131P^ CRISPR mice, using sgRNA (CTGTCGATCTTCCTGCTGCC) and oligo donor (CTGTTGCTGCTGCTTCAGGTTTATCATTGGAATACCGTTTCAGATGTCAGCCAGCCAGCACCATGGCAGCAGGAAGATCGACAGGATGCAGAGGAAGACGGgtgagtctcccaaagactggga). Sex was not recorded for mice that were sampled at E19.5, P2 and P3. Experiments using adult mice were performed with a combination of both males and females between 8 and 12 weeks of age. Mice were housed in a temperature and humidity controlled specific pathogen free facility with a 12 h:12 h day night cycle. The WEHI Animal Ethics Committee approved all experiments in accordance with the NHMRC Australian code for the care and use of animals for scientific purposes.

### Linkage analysis

We mapped the chromosomal location of the *Plt15* mutation by mating affected mice to 129/Sv *Mpl*^−/−^ mice to produce N_2_ (backcross) and F_2_ (intercross) generations. A genome-wide scan using 20 N_2_ mice with the highest platelet counts (287 ± 74 × 10^6^ per mL, compared with 133 ± 75 × 10^6^ per mL for the overall population, Fig. 1a) localized the mutation to a region of chromosome 8 between *D8Mit242* and *D8Mit139* and linkage to this region was then refined. Analysis of the F_2_ population revealed a significant reduction in the frequency of mice homozygous for *C57BL/6* alleles in this interval (e.g., *D8Mit200* 3/81 F_2_ mice homozygous *C57BL/6*, p = 2.2 × 10^−5^ *χ*^2^-test), suggesting the *Plt15* mutation results in recessive lethality. The refined 2.01 Mb interval contained 31 annotated genes, only five of which appeared to be expressed both in the hematopoietic system and during embryogenesis (http://biogps.gnf.org/): *Dead box proteins 19a* and *19b* (*Ddx19a* and *Ddx19b*), *Ring finger and WD repeat domain 3* (*Rfwd3*), *Mixed lineage kinase domain like* (*Mlkl*), and *WD40 repeat domain 59* (*Wdr59*). Sequencing identified a single mutation, an A to T transversion in *Mlkl* that was heterozygous in all mice with an elevated platelet count.

### Reagents

Antibodies; Rat-anti mRIPK3 and rat anti-mMLKL 8F6 (selected for affinity to residues 1–30 of mouse MLKL) and rat anti-MLKL 3H1^4^ (MLKL brace region) were produced in-house. Anti-Pro Caspase 8 (#4927) and GAPDH (#2113) were purchased from Cell Signaling Technology. Anti-mouse MLKL pS345 (ab196436) and anti-Actin (ab5694) were purchased from Abcam. Anti-VDAC (AB10527) was purchased from Millipore. Fc-hTNF was produced in house and used at a final concentration of 100 ng per mL. Recombinant mouse IFN-γ and β were purchased from R&D Systems (Minneapolis, MN, USA) Q-VD-OPh and zVAD-fmk were purchased from MP Biomedicals (Seven Hills, NSW, Australia). Smac mimetic also known as Compound A, and the caspase inhibitor IDN-6556 were a gift from TetraLogic (Malvern, PA, USA). Propidium iodide, doxycycline, and bafilomycin were purchased from Sigma-Aldrich (Castle Hill, NSW, Australia).

### Cell line generation and culture

Primary mouse dermal fibroblasts were prepared from skin taken from the head and body of E19.5 pups delivered by C-section or from the tails of adult mice^69^. Primary MDFs were immortalized by stable lentiviral transduction with SV40 large T antigen. Immortalized MDFs were stably transduced with exogenous mouse MLKL cloned into the pFTRE 3 G vector, which was generated by Toru Okamoto, and allows doxycycline- inducible expression as described^4^. The following oligonucleotides were used for the assembly of constructs;

*mMlkl* fwd; 5′-CGCGGATCCGCGCCACCatggataaattgggacagatcatcaag-3′,

*mMlkl* rev; 5′-CGGAATTCttacaccttcttgtccgtggattc-3′,

N-FLAG *mMlkl* fwd; 5′-CGCGGATCCAA gccacc atg gcg cgc cag gac-3′

N-FLAG *mMlkl* rev; 5′-CGCGGATCC tta cac ctt ctt gtc cgt gga ttc-3′

*mMlkl* D139V fwd; 5′-gaagatcgacaggTtgcagaggaagac-3′

*mMlkl* D139V rev; 5′-gtcttcctctgcaAcctgtcgatcttc-3′

mMlkl S131P fwd; 5′-gccagcctgcaCcctggcagcag-3′

mMlkl S131P rev; 5′-ctgctgccaggGtgcaggctggc-3′

Cells were maintained in culture as previously described^44^. 4-hydroxy-tamoxifen regulated HOXA9 immortalised Factor Dependent Myeloid cells were generated from mouse E14.5 fetal liver cells and cultured as described previously^70^.

### Cell death assays

Flow Cytometry based cell death assays were performed using 5 × 10^4^ MDFs per well in 24 or 48-well tissue culture plates^4^. Doxycycline (20 ng per mL) was added together with death stimuli. Fc-hTNF was produced in house and used at 100 ng per mL, Compound A Smac mimetic and IDN6556 were used at 500 nM and 5 μM respectively. zVAD-fmk and QVD-OPh were used at 25 and 10 μM respectively. Mouse and human interferons γ and β were used at 30 ng per mL, PS341 and MG132 at 2 and 200 nM respectively and Bafilomycin at 300 nM. For Incucyte automated imaging, MDFs were plated at a density of 8 × 10^3^ cells per well of a 96-well plate and permitted to attach for 3 h. FDMs were plated at a density of 5 and 10 × 10^3^ cells per well of a 48-well plate. 0.2 μg per mL propidium iodide was in media alongside stimuli as indicated. Images were recorded at intervals of 1 and 2 h using an IncuCyte S3 and numbers of PI positive cells per mm^2^ at each time point quantified and plotted using IncuCyte S3 software.

### MLKL turn-over assays

5 × 10^4^ MDFs per well were plated in 24-well tissue culture plates and allowed to settle. Doxycycline (20 ng per mL) +/− TNF, Smac Mimetic and IDN6556 was added. After 15 h, ‘no dox’ and ‘0’ wells were harvested. Media was removed from remaining wells and cells were washed with PBS and fresh media containing IDN6556 was re-added. Wells were then harvested 2, 4, 6, 8, and 24 h from this point. Cells were harvested by direct lysis in reducing SDS-PAGE loading buffer.

### MLKL protection assays

5 × 10^4^ MDFs per well were plated in 24-well tissue culture plates and allowed to settle. Doxycycline (20 ng per mL) was added. After 18 hrs, ‘no dox’ and ‘T_0_’ samples were harvested. Media was removed and cells washed before addition of fresh media containing TSI or IDN alone for 3 h. Cells were washed again and media restored with IDN6556 alone (UT), or IDN6556 + inhibitor (MG132 (200 nM), PS341 (10–40 nM), Chloroquine (50 μM), Bafilomycin (300 nM), Ca-074 Me (20 μM), TLCK (100 μM) and AEBSF (100 μM)) for a further 21 h. Cells were harvested by direct lysis in reducing SDS-PAGE loading buffer.

### UBA pull downs

2 × 10^6^ MDFs stably transduced with doxycycline inducible N-FLAG-mMLKL^WT^ or N-FLAG-mMLKL^D139V^ expressing constructs were seeded and settled O/N before stimulation with 1 μg per mL doxycycline +/− TSI for 5 hrs. Cells were lysed in Urea-based UBA-pull down buffer, ubiquitylated proteins enriched and Usp21-treated as described previously^71^.

### Transmission electron microscopy

Murine dermal fibroblasts prepared from mice of the indicated genotypes were untreated or stimulated with the indicated agents for the indicated hours. Then, cells were fixed with 2% glutaraldehyde in 0.1 M phosphate buffer, pH 7.4, postfixed with 2% OsO_4_, dehydrated in ethanol, and embedded in Epok 812 (Okenshoji Co.). Ultrathin sections were cut with an ultramicrotome (ultracut N or UC6: Leica), stained with uranyl acetate and lead citrate, and examined with a JEOL JEM-1400 electron microscope. The viability of a portion of these cells was determined by measuring LDH release as described previously^72^.

### Mouse histopathology

Caesarian-sectioned E19.5 and Day P2/3 pups were euthanized by decapitation and fixed in 10% buffered formalin. Five-micrometers coronal sections were taken at 200-μm intervals for the full thickness of the head, 5-μm sagittal sections were taken at 300-μm intervals for the full thickness of the body. A thorough examination of these sections was performed by histopathologists Aira Nuguid and Tina Cardamome at the Australian Phenomics Network, Melbourne. Findings were confirmed by Veterinary Pathologist Prof. John W. Finney, SA Pathology, Adelaide and clinical Pathologist Prof. Catriona McLean, Alfred Hospital, Melbourne.

### Measurement of relative thymic cortical thickness

Representative images of thymus sections were analysed to determine relative cortical thickness using ImageJ. Briefly, medullary areas were identified on the basis of H and E staining and removed from the larger thymus structure using the Image J Image Calculator function to isolate the cortical region. The thickness of the cortical region, defined by the radius of the largest disk that can fit at a pixel position, was determined using the Local Thickness plugin in ImageJ (http://www.optinav.info/Local_Thickness.htm).

### Immunohistochemistry

Following terminal blood collection, P0 and P3 pups were fixed for at least 24 h in 10% buffered formalin and paraffin embedded before microtomy. Immunohistochemical detection of cleaved caspase 3 (Cell Signaling Technology #9661) and CD45 (BD) was performed as described previously^23^.

### Cytokine quantification

All plasma was stored at −80 °C prior to cytokine analyses. Cytokines were measured by Bioplex Pro mouse cytokine 23-plex assay (Bio- Rad #M60009RDPD) according to manufacturer’s instructions. When samples were designated ‘<OOR’ (below reference range) for a particular cytokine, they were assigned the lowest value recorded for that cohort (as opposed to complete exclusion or inclusion as ‘zero’ which would artificially inflate or conflate group averages respectively). Values are plotted as fold change relative to the mean value for the *Wt/Wt* samples, and *p* values were calculated in Microsoft Excel using a two-tailed TTEST, assuming unequal variance. Data is only shown for cytokines that displayed statistically significant differences between genotypes at either of or both day E19.5 and day P3.

### Hematological analysis

Blood was collected from P0 and P3 pups into EDTA coated tubes using heparinized glass capillary tubes from the neck cavity immediately after decapitation. After centrifugation at 500 G for 5 min, 5–15 μL of plasma was carefully removed and this volume was replaced with PBS. Blood cells were resuspended and diluted between 8–20-fold in DPBS for automated blood cell quantification using an ADVIA 2120 hematological analyzer within 6 h of harvest. Blood was collected from adult mice retro-orbitally into tubes containing EDTA and analyzed using an ADVIA120 automated hematological analyzer (Bayer).

### Transplantation studies

Donor bone marrow or fetal liver cells were injected intravenously into recipient *C57BL/6-CD45*^*Ly5.1/Ly5.2*^ mice following 11 Gy of gamma-irradiation split over two equal doses. Recipient mice received neomycin (2 mg per mL) in the drinking water for 4 weeks. Long term capacity of stem cells was assessed by flow cytometric analysis of donor contribution to recipient mouse peripheral blood and/or hematological organs up to 6 months following engraftment. Recovery from cytotoxic insult was assessed by automated peripheral blood analysis at regular times following treatment of mice with 150 mg per kg 5-fluorouracil (5-FU).

### Flow cytometry

To analyze the contribution of donor and competitor cells in transplanted recipients, blood cells were incubated with a combination of the following antibodies: Ly5.1-PE, Ly5.2-FITC, Ly5.2-biotin or Ly5.2 PerCPCy5.5 (antibodies from Becton Dickenson, Ca). If necessary, cells were incubated with a streptavidin PECy5.5 (BD), mixed with propidium iodide (Sigma) and analysed on a LSRI (BD Biosciences) flow cytometer. To analyse the stem- and progenitor cell compartment, bone marrow cells were incubated with biotinylated or Alexa700 conjugated antibodies against the lineage markers CD2, CD3, CD4, CD8, CD34, B220, CD19, Gr-1, and Ter-119. For stem and progenitor cell detection antibodies against cKit, Sca-1, CD48, AnnexinV, CD105, FcγRII/III or CD135 in different combinations (see antibody list for details). Finally FluoroGold (AAT Bioquest Cat#17514) was added for dead cell detection. Cells were then analysed on LSRII or Fortessa1 (BD Biosciences) flow cytometers.

### Reactive oxygen species (ROS) detection

ROS was detected by using Chloromethyl-H_2_DCFDA dye according to the manufacturer’s instructions (Invitrogen Cat#C6827). In brief, bone marrow cells were loaded with 1 μM Chloromethyl-H_2_DCFDA for 30 min at 37 °C. Loading buffer was then removed, and cells were placed into 37 °C StemPro-34 serum free medium (ThermoFisher Cat#10639011) for a 15-min chase period. After incubation cells were placed on ice and stained with surface antibodies suitable for FACS analysis. Cells were analysed using a LSRII flow cytometer (Becton Dickinson).

### E14.5 fetal liver colony forming assays

1 × 10^4^ fetal liver cells were plated as 1 mL cultures in 35 mm Petri dishes in DMEM containing 10% FCS, 0.3% agar and 10^4^ U per mL GM-CSF. IFN-β was added to the cultures in increasing concentrations from 0 to 30 ng per mL. Colony formation was scored after 7 days of incubation at 37 °C, fully humidified with 10% CO_2_.

### Quantitative PCR

RNA was prepared using Trizol (Invitrogen) according to the manufacturer’s instructions and 10 μg was used for first strand cDNA synthesis using SuperScript II (Life Technologies). 0.5 μg of cDNA was then used in a TaqMan PCR reaction with Universal PCR mastermix and murine Mlkl (Mm1244222_n1) and GAPDH (Mm99999915_m1) Taqman probes (ThermoFisher) on an ABI 7900 Fast Real-Time PCR instrument (Applied Biosystems). *Mlkl* expression relative to GAPDH control was determined using SDS version 2.3 program (Applied Biosystems) and expressed as ΔCT values.

### Statistics (mouse and cell-based assays)

Please consult figure legends for description of error bars used. All data points signify independent experimental repeats, and/or biologically independent repeats. All *p* values were calculated in Microsoft Excel or Prism using an unpaired, two-tailed *t*-test, assuming unequal variance and not adjusted for multiple comparison. Asterices signify that *p* ≤ 0.05 (*), *p* ≤ 0.01(**) or *p* ≤ 0.005 (***). All comparisons were made between *Mlk*^*Wt/Wt*^ and *Mlk*^*D139V/D139V*^ groups only (with the exception of data derived from adult mice, which were comparisons between *Mlk*^*Wt/Wt*^ and *Mlk*^*Wt/D139V*^ groups only.

### Whole-exome sequencing

DNA from CRMO probands and their family members (when available) was purified from saliva or blood and prepared for whole-exome sequencing (WES). The samples underwent WES at several different times, enriched using the Agilent SureSelect Human All Exon V4, V5 or V6 + UTR (Agilent Technologies) before sequencing at either Otogenetics, Inc (Atlanta, GA), Beckman Coulter Genomics (Danvers, MA), or at the University of Iowa Genomics Core (Iowa City, IA). The fastq files were quality-checked and processed to vcf format as described^73^. Variants for all samples were called together using GATK’s Haplotype Caller^74^ and were recalibrated and hard-filtered in GATK as described^73^. Variants were annotated with minor allele frequencies (MAFs) from 1000 genomes^75^, ExAC and gnomAD^48^ and with information regarding the effect of each variant using SNPSift/SNPEff^76^. The databases used for annotation were dbNSFP2.9^77^ (for MAFs) and GRCh37.75 for protein effect prediction.

### Ancestry determination

Ancestry was determined for each CRMO proband using the LASER software package^78^. A vcf file including ten probands at a time was uploaded to the LASER server and the TRACE analysis was selected using the Worldwide panel. For probands with indeterminate ancestry using the Worldwide panel, the European and Asian panels were used. Principal component values for each proband were plotted using R Statistical Software and the code provided in the LASER package.

### MLKL variant quantification

1000 Genomes: Vcf files from 1000 genomes were annotated and filtered as described previously^79^. Values for MLKL variants rs35589326 (S132P), rs34515646 (R146Q), and rs144526386 (G202V) as well as all MLKL coding variants were queried and tabulated for allele and genotype count for participants of all ancestry (*n* = 2504), and for those of European ancestry (*n* = 503). Compound heterozygous variants were evident due to the phasing of all variants in the 1000 genomes dataset. *CRMO:* Allele and genotype counts for all MLKL coding variants were tabulated in probands of European ancestry (*n* = 101) and for all probands (*n* = 128). Compound heterozygous variants were identified using parental sequence data. *AS:* DNA from all subjects in AS cohort were genotyped using the Illumina CoreExome chip following standard protocols at the Australian Translational Genomics Centre, Princess Alexandra Hospital, Brisbane. Bead intensity data was processed and normalized for each sample and genotypes called using the Illumina Genome Studio software. All the samples listed in the table have passed quality control process^80^. GB: Genotyping was performed in an ISO15189-accredited clinical genomics facility, Australian Translational Genomics Centre (ATGC), Queensland University of Technology. All samples were genotyped by Illumina HumanOmniExpress (OmniExpress) BeadChip^81^. QUT controls: A collection of healthy control data of verified European ancestry from various cohort studies, complied by the Translational Genomics Group, QUT and typed on an Illumina CoreExome microarray. Includes data from The UK Household Longitudinal Study, led by the Institute for Social and Economic Research at the University of Essex and funded by the Economic and Social Research Council. The survey was conducted by NatCen and the genome-wide scan data were analysed and deposited by the Wellcome Trust Sanger Institute. University of Essex. Institute for Social and Economic Research, N. S. R., Kantar Public. Understanding Society: Waves 1–8, 2009–2017 and Harmonised BHPS: Waves 1–18, 1991–2009. [data collection]. 11th Edition. UK Data Service., (2018).

### Patient recruitment

All genomic data was derived from patients recruited with consent as described previously^48,80,81^, and with the approval of human ethics review boards of all Institutes that participated in human genetics studies; University of Iowa Carver College of Medicine, Queensland University of Technology, Australian National University, Shanghai Renji Hospital, JiaoTong University of Shanghai, The Hospital for Sick Children and the University of Toronto, University of Sydney, Australian Institute of Sport, University of Freiburg, Princess Alexandra Hospital, Memorial Hermann Texas Medical Centre, The University of Queensland, Oregon Health and Science University), Groupe Française d’Etude Génétique des Spondylarthrites (GFEGS) and the University of Oxford.

### Statistical analysis (human data)

Statistical comparisons were performed at the level of allele frequency or the level of compound heterozygote sample frequency using either a Fisher’s exact test or a Chi-Squared test with Yates correction as specified under each table. Compound heterozygous variants were quantified and compared at the individual rather than the allelic level, where individuals with and without qualifying variants were compared at the allelic level.

### Web resources

gnomAD – https://gnomad.broadinstitute.org/

http://asia.ensembl.org

OrthoDB - https://www.orthodb.org

CADD - https://cadd.gs.washington.edu/

Clustal Omega - https://www.ebi.ac.uk/Tools/msa/clustalo/

WEBLOGO - https://weblogo.berkeley.edu/logo.cgi

Missense Tolerance Ratio (MTR) Gene Viewer - http://biosig.unimelb.edu.au/mtr-viewer

UK biobank - https://www.ukbiobank.ac.uk

Understanding Society - https://www.understandingsociety.ac.uk/

### Reporting summary

Further information on research design is available in the Nature Research Reporting Summary linked to this article.

## Supplementary information

Supplementary Information

Reporting Summary

## Data Availability

The biological tools generated for *MLKL* during this study are available from the corresponding authors on reasonable request. *MLKL* gene variants in CRMO can be accessed from Harvard Dataverse, V1 [10.7910/DVN/BL3SXQ]. The source data underlying Figs. 1a, d, e–g, 2a–f, 3a–k, 4a–f, 5c, d and Supplementary Figs. 1a, b, 2a–h, 3a, c, d–f, 4b, d–j are provided as a Source Data file. Source data are provided with this paper.

## Source data

Source Data
