# Supplementary material for: A missense mutation in the MLKL brace region promotes lethal neonatal inflammation and hematopoietic dysfunction
Source: Nat Commun. 2020 Jun 19;11:3150. doi: 10.1038/s41467-020-16819-z (PMC7305203; doi:10.1038/s41467-020-16819-z)
Supplement: Supplementary file 1 — Supplementary Information [file 41467_2020_16819_MOESM1_ESM.pdf]

Supplementary Information for Hildebrand and Kauppi et al (2020) 'A missense mutation in the MLKL brace region leads to lethal neonatal inflammation and hematopoietic dysfunction'.

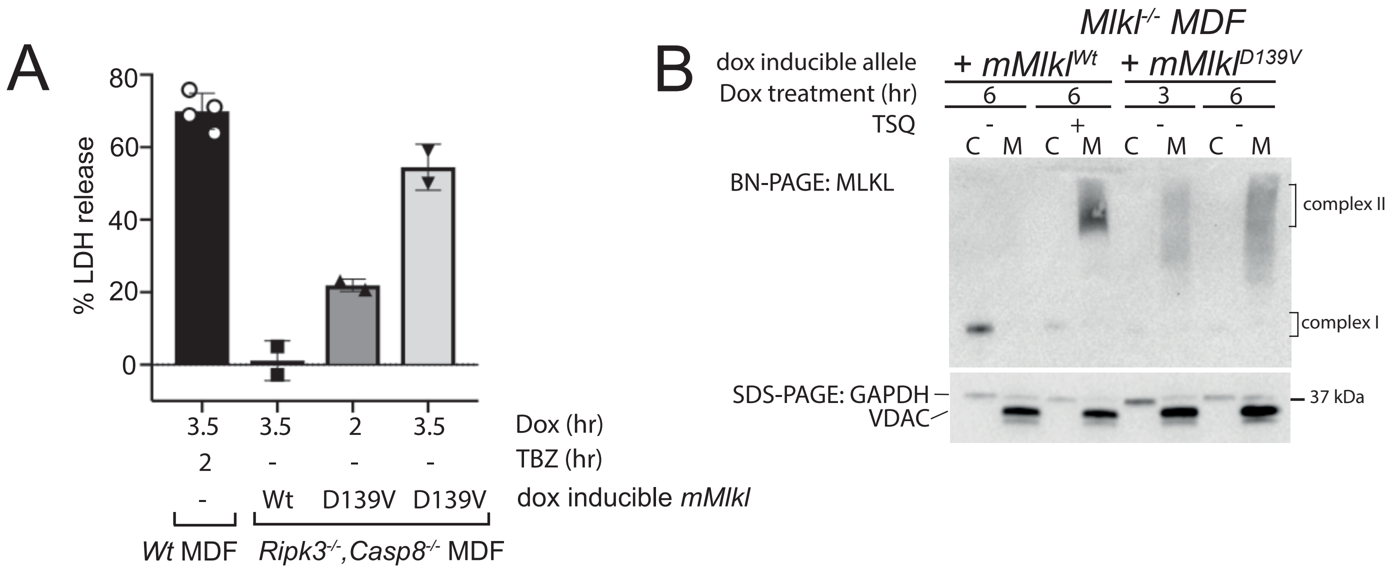

Hildebrand and Kauppi *et al*, Supplementary Figure 1. (A) Viability of non-transduced Wt MDFs or *RIPK3*$^{-/-}$, *Caspase8*$^{-/-}$ MDFs expressing dox-inducible *Mlkl*^{Wt} or *Mlkl*^{D139V} was monitored by measuring LDH release at the indicated time points post addition of TNF (T), Birinpant (B) and ZVAD-fmk (Z) or doxycycline. These conditions correspond to those used for TEM analyses **(Figure 1G)**. Mean ± range for 2 independent experiments using *Ripk3*$^{-/-}$,*Casp8*$^{-/-}$ MDFs, or mean ± SD for 4 independent experiments (Wt MDFs) plotted. **(B)** MLKL^{D139V} spontaneously forms membrane-associated high molecular weight complexes (as assessed by Blue Native (BN) PAGE) in the absence of extrinsic necroptotic stimuli when expressed in mouse dermal fibroblasts. C; cytoplasmic fraction, M; crude membrane fraction, TSI; TNF, Smac-mimetic and IDN6556. Image representative of 3 similar independent experiments.

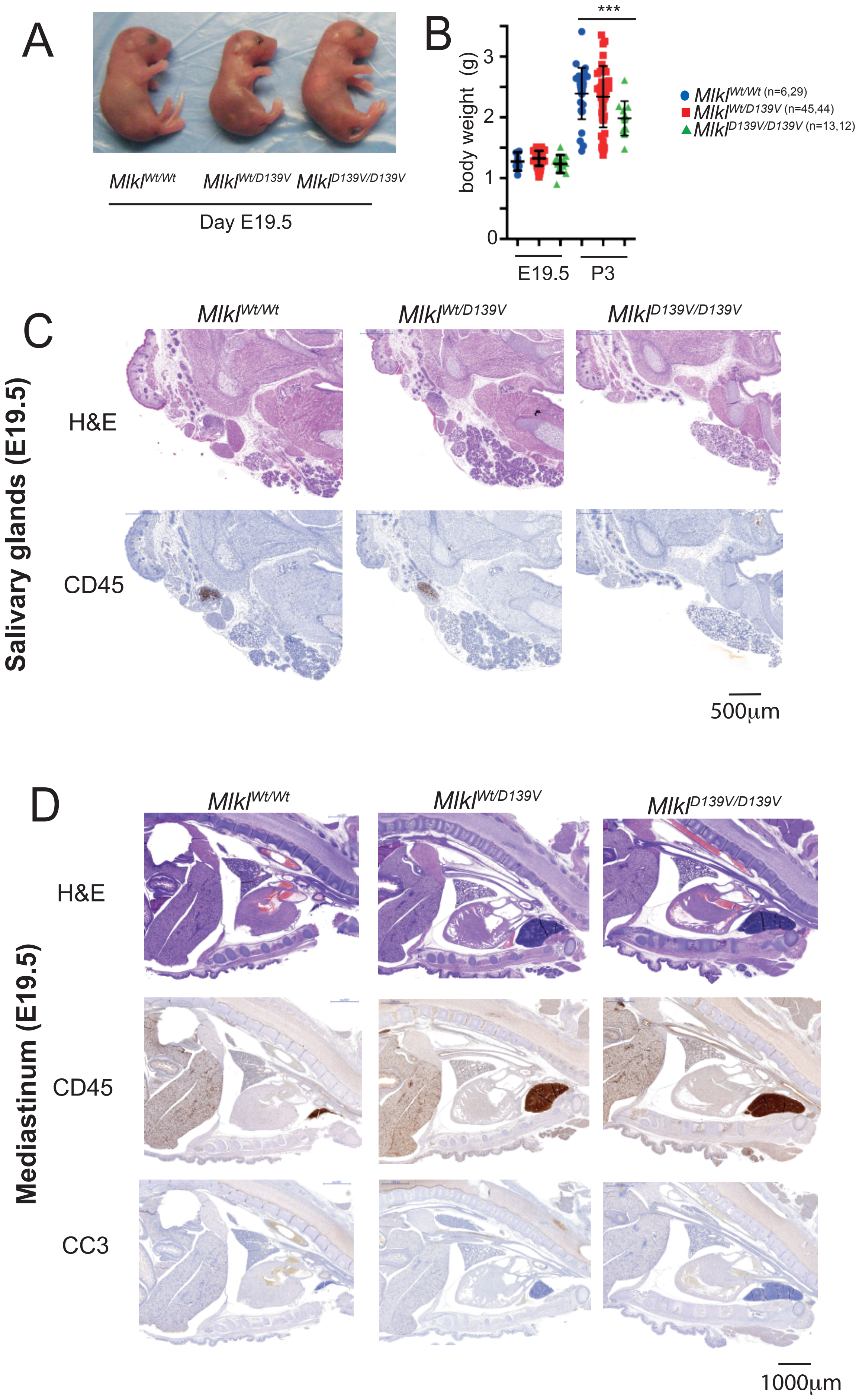

Hildebrand and Kauppi *et al*, Supplementary Figure 2

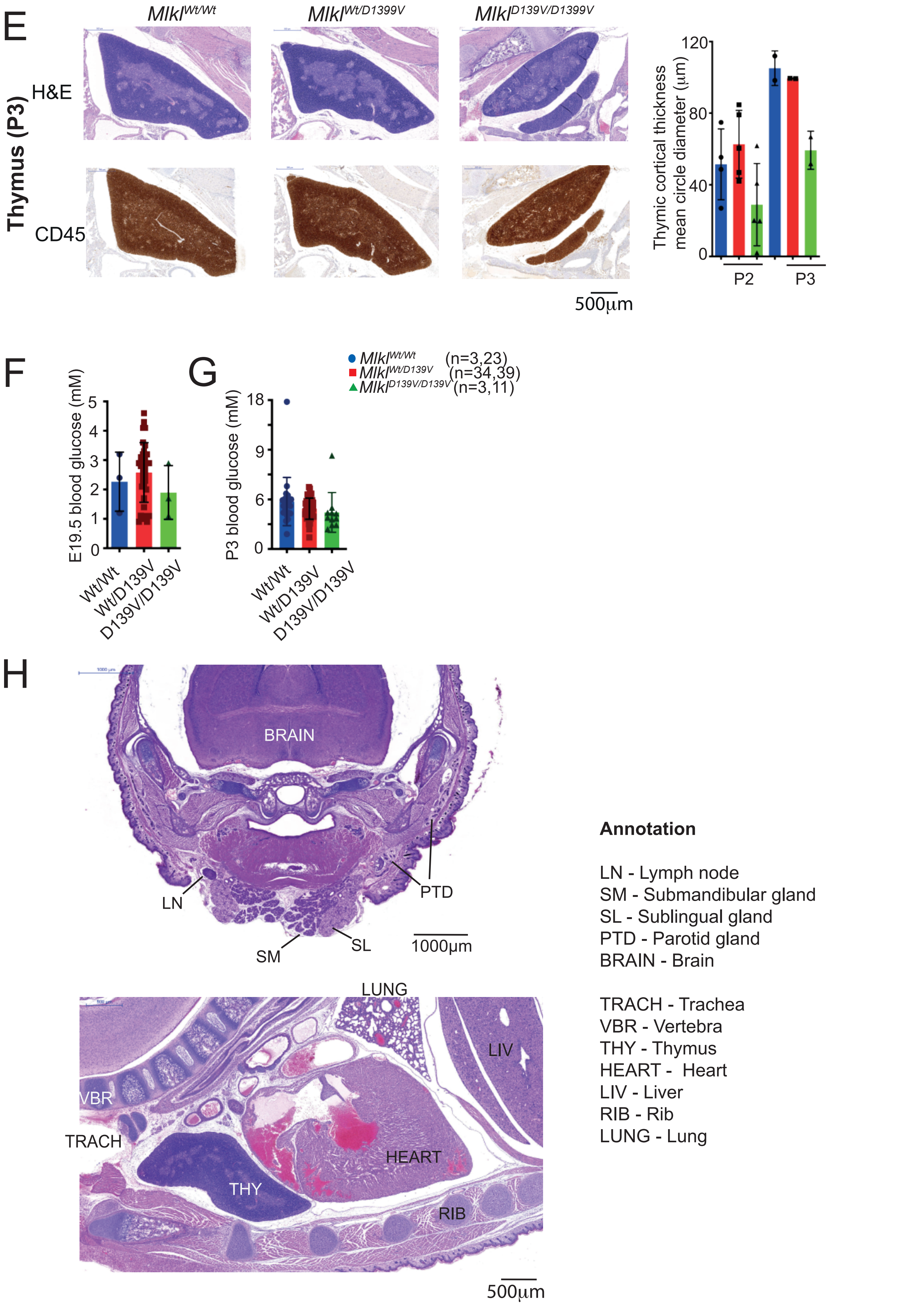

Hildebrand and Kauppi *et al*, Supplementary Figure 2

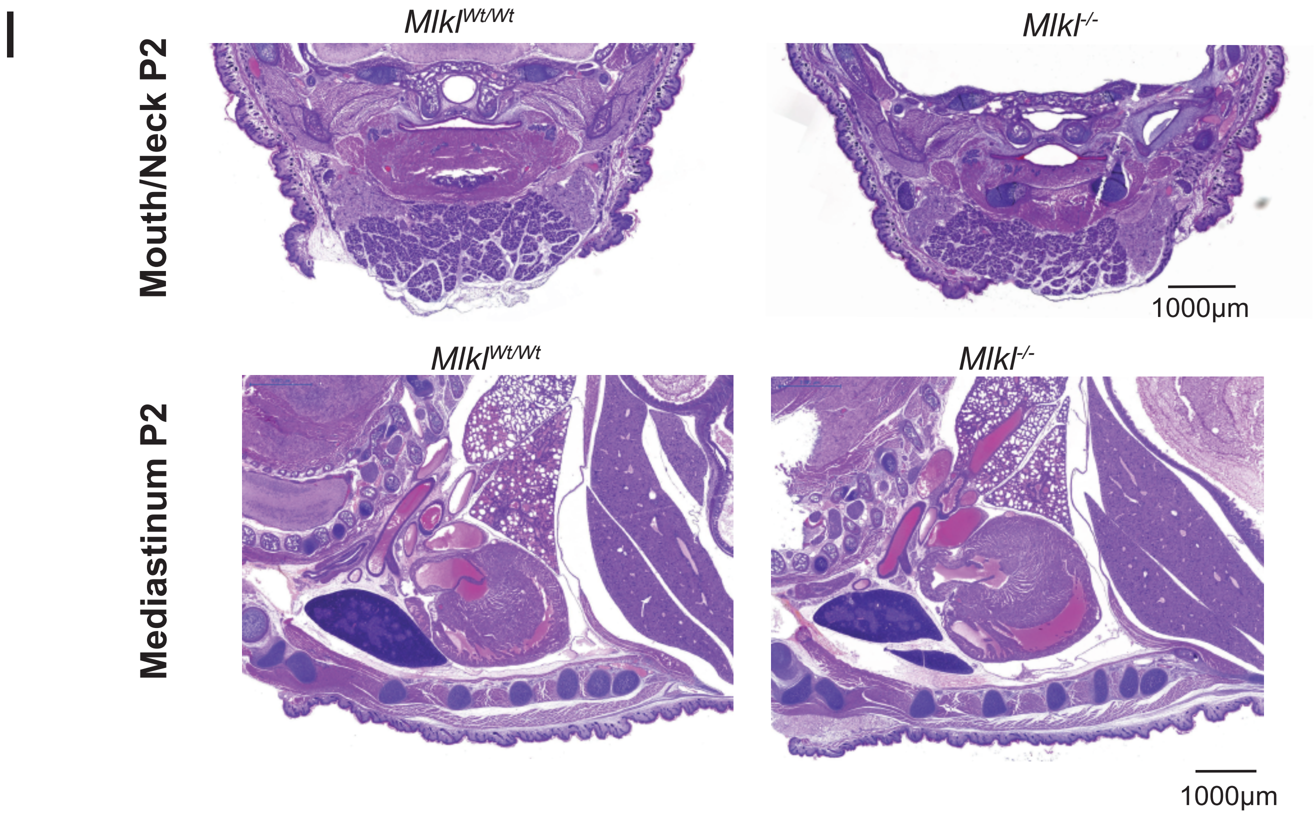

Hildebrand and Kauppi *et al*, Supplementary Figure 2. **(A)** Macroscopic appearance of E19.5 pups of indicated genotypes after Caesarean delivery. **(B)** Body weights of *Mlkl*$^{Wt/Wt}$, *Mlkl*$^{Wt/D139V}$, *Mlkl*$^{D139V/D139V}$ mice at E19.5 and postnatal Day 3. **(C)** Serial mandible sections from E19.5 pups stained with H&E and anti-CD45. **(D)** H&E and anti-CD45 or cleaved caspase-3 (CC3) stained section of E19.5 mediastinum. **(E)** Serial sections of thymi from postnatal day 3 pups stained with H&E and anti-CD45 and quantification of thymic cortical thickness for n = 5 P2 pups per genotype, and n = 2 P3 pups per genotype. **(F-G)** Blood glucose measured at E19.5 and postnatal day 3 (non-fasting). **(H)** Anatomical annotation of head and mediastinum of postnatal day 2 *Mlkl*$^{Wt/Wt}$ pup. **(I)** Coronal section of postnatal day 2 pup mouth/neck region and mediastina stained with H&E. For **(B), (E), (F)** and **(G)**, each symbol represents one independent pup sampled; *Mlkl*$^{Wt/Wt}$ – blue circles, *Mlkl*$^{Wt/D139V}$- red squares, *Mlkl*$^{D139V/D139V}$- green triangles, with bar height and error bars representing mean ± SD repectively, or range when n = 2. Precise number of pups sampled as indicated. Images presented in **(C)**, **(E) (H)** and **(I)** are representative of images taken for at least 3 pups examined per genotype with similar characteristics. * $p \leq 0.05$, ** $p \leq 0.01$, ***$p \leq 0.005$ calculated using an unpaired, two tailed t-test.

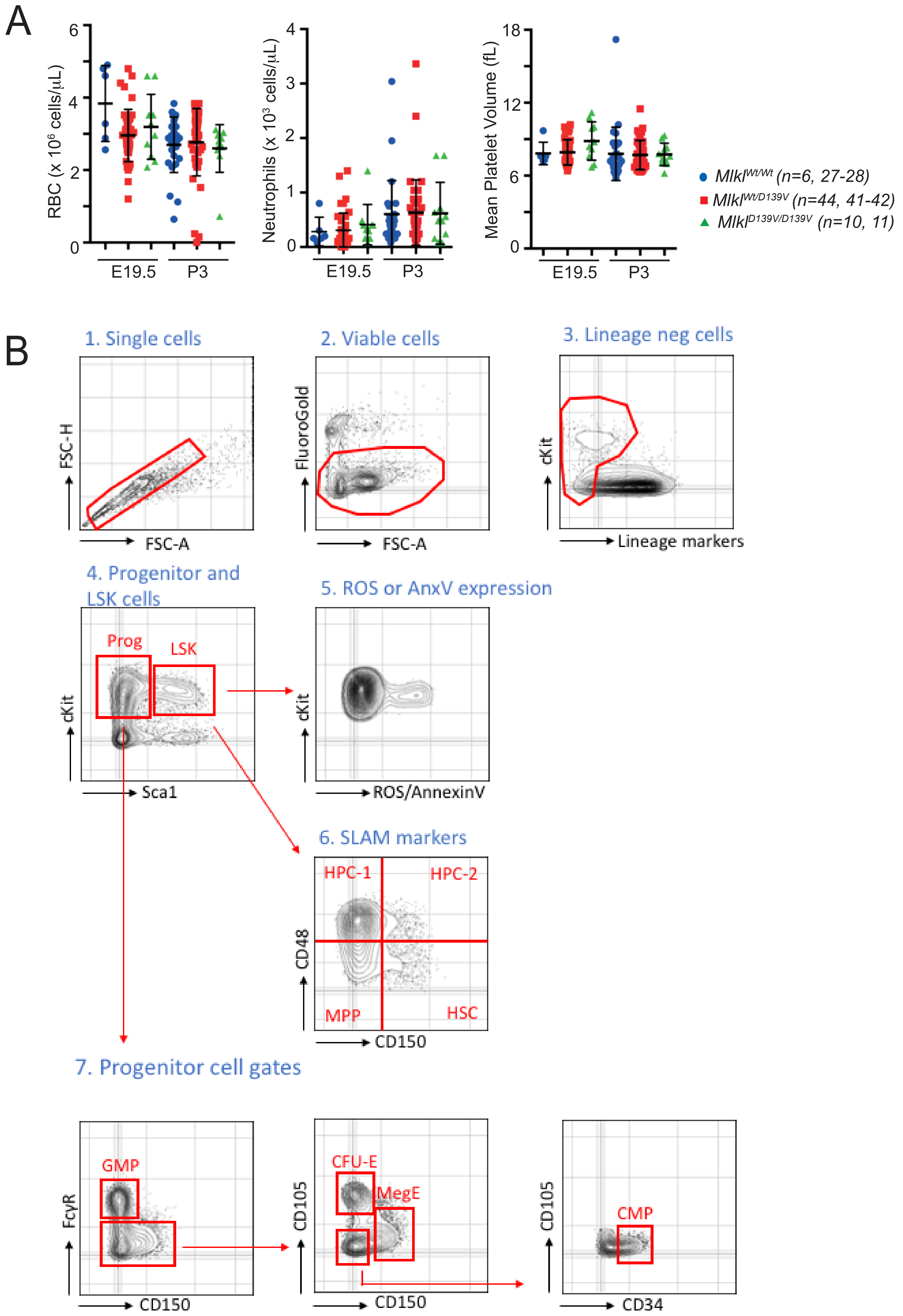

Hildebrand and Kauppi *et al*, Supplementary Figure 3

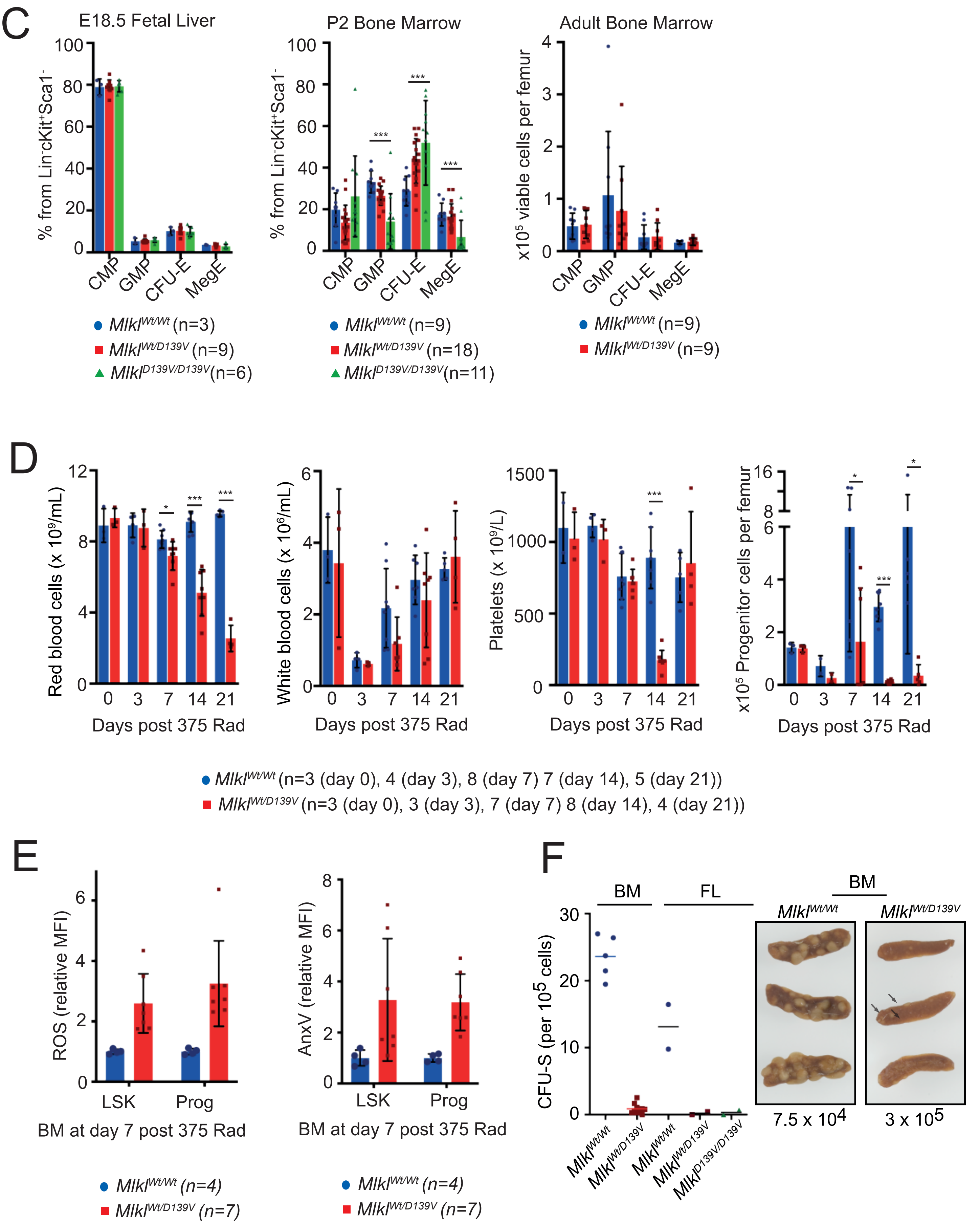
C
E18.5 Fetal Liver
P2 Bone Marrow
Adult Bone Marrow
% from Lin-cKit+Sca1-
x10^5 viable cells per femur
CMP
GMP
CFU-E
MegE
***
Mlkl^Wt/Wt (n=3)
Mlkl^Wt/D139V (n=9)
Mlkl^D139V/D139V (n=6)
Mlkl^Wt/Wt (n=9)
Mlkl^Wt/D139V (n=18)
Mlkl^D139V/D139V (n=11)
Mlkl^Wt/Wt (n=9)
Mlkl^Wt/D139V (n=9)
D
Red blood cells (x 10^9/mL)
White blood cells (x 10^6/mL)
Platelets (x 10^9/L)
x10^5 Progenitor cells per femur
Days post 375 Rad
Mlkl^Wt/Wt (n=3 (day 0), 4 (day 3), 8 (day 7) 7 (day 14), 5 (day 21))
Mlkl^Wt/D139V (n=3 (day 0), 3 (day 3), 7 (day 7) 8 (day 14), 4 (day 21))
E
ROS (relative MFI)
AnxV (relative MFI)
LSK
Prog
BM at day 7 post 375 Rad
Mlkl^Wt/Wt (n=4)
Mlkl^Wt/D139V (n=7)
F
BM
FL
CFU-S (per 10^5 cells)
Mlkl^Wt/Wt
Mlkl^Wt/D139V
Mlkl^D139V/D139V
7.5 x 10^4
3 x 10^5

Hildebrand and Kauppi *et al*, Supplementary Figure 3. (A) Numbers of red blood cells (RBC), neutrophils and mean platelet volume in the peripheral blood of E19.5 and P3 pups. **(B)** Gating strategy used for enumeration of Progenitor subsets, LSK subsets, ROS or Annexin V positive HSC subsets in **Figures 3 (D-G)** and **Supp. Fig. 3 (C-E)**. **(C)** Common myeloid progenitors (CMP, Lineage$^-$ IL7Rα$^-$ Sca1$^-$ cKit$^+$ CD34$^+$ FcγRII/III$^-$), granulocyte-macrophage progenitors (GMP, Lineage$^-$ IL7Rα$^-$ cKit$^+$ Sca1$^-$ CD150$^-$ Endoglin$^-$ FcγRII/II$^+$), Colony-forming units-erythroid (CFU-E, Lineage$^-$ IL7Rα$^-$ cKit$^+$ Sca1$^-$ CD150$^-$ FcγRII/III$^-$ Endoglin^{hi}), and megakaryocyte-erythroid progenitors (MegE, Lineage$^-$ IL7Rα$^-$ Sca1$^-$ cKit$^+$ CD150$^+$ Endoglin^{low} FcγRII/III$^-$) in E18.5 fetal livers, P2 bone marrow cells and adult bone marrow, presented as a percent from Lin$^-$ cKit$^+$ Sca1$^-$ cell fractions. Mean ± SD, n=3-6 (E18.5), n=9-11 (P2 BM), n=9 (adult BM) per genotype. **(D)** Recovery of red blood cells, white blood cells, platelets and bone marrow progenitor cells (Lineage$^-$ Sca$^-$ Kit$^+$) in *Mlkl$^{Wt/Wt}$* (blue circles) and *Mlkl$^{Wt/D139V}$* (red squares) mice following 375 Rad whole body irradiation. **(E)** Relative amount of ROS and AnnexinV in LSK and progenitor cells was determined 7 days post irradiation. **(F)** Bone marrow (BM) or fetal liver (FL) cells (7.5x10^4 - 3x10^5) from mice of the indicated genotypes (*Mlkl$^{Wt/Wt}$*, *Mlkl$^{Wt/D139V}$* or *Mlkl$^{D139V/D139V}$*) were transplanted into lethally irradiated recipients and spleens were removed for enumeration of CFU-S after 8 days. Mean ± SEM from 2-8 donors. Spleens taken from recipients of *Mlkl$^{Wt/Wt}$* or *Mlkl$^{Wt/D139V}$* bone marrow (7.5 x10^4 or 3.0 x10^5 cells transplanted respectively) were photographed to detail the size and number of colonies. *Mlkl$^{Wt/D139V}$* cells generated very small colonies at low frequency (arrows). For **(A), (C), (D)** and **(E)**, each symbol represents one independent pup sampled; *Mlkl$^{Wt/Wt}$* – blue circles, *Mlkl$^{Wt/D139V}$*- red squares, *Mlkl$^{D139V/D139V}$*- green triangles, with bar height and error bars representing mean ± SD repectively, or range when n = 2. Precise number of pups sampled as indicated. * $p \le 0.05$, ** $p \le 0.01$, ***$p \le 0.005$ calculated using an unpaired, two tailed t-test.

A

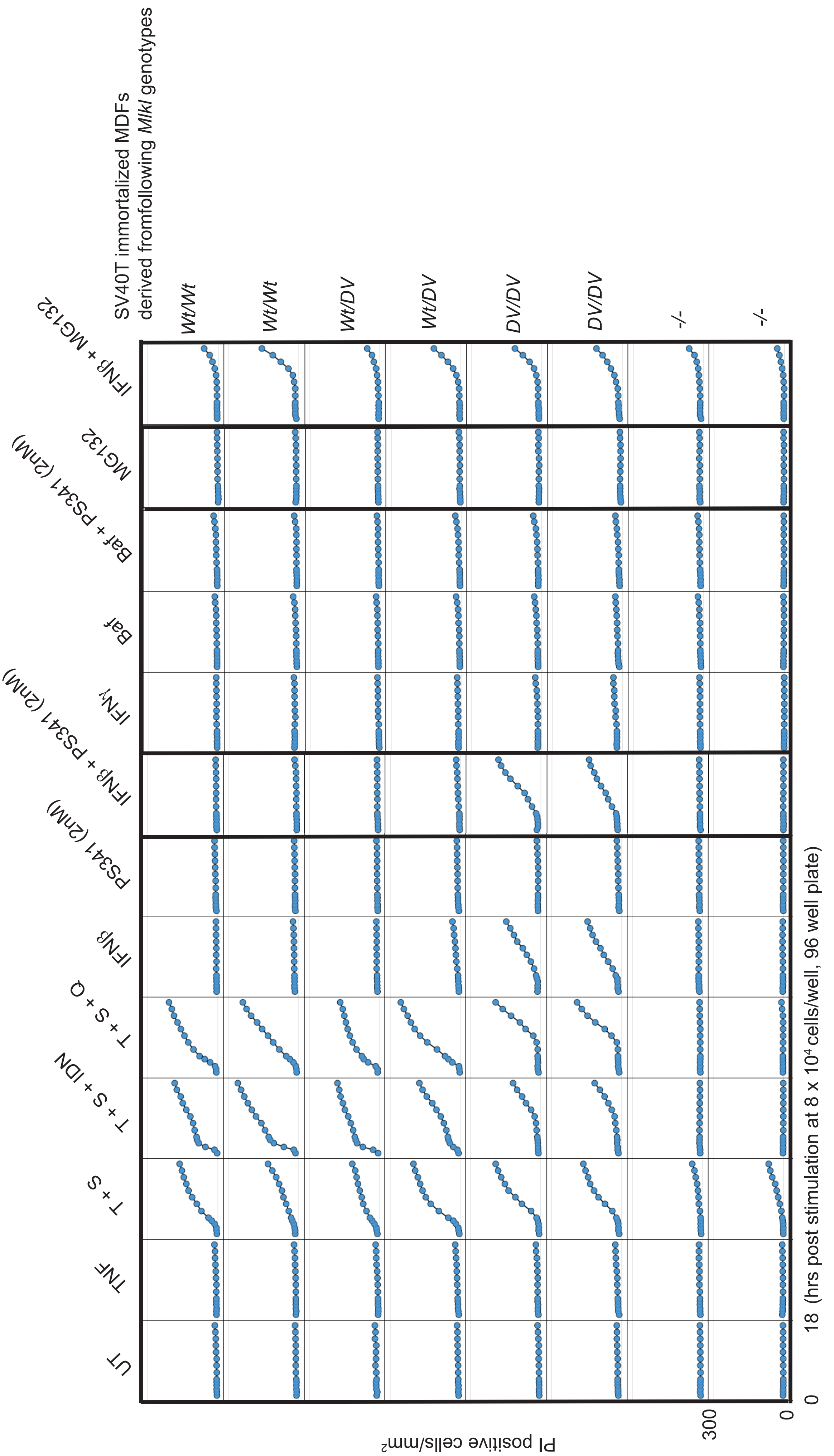

Hildebrand and Kauppi *et al*, Supplementary Figure 4

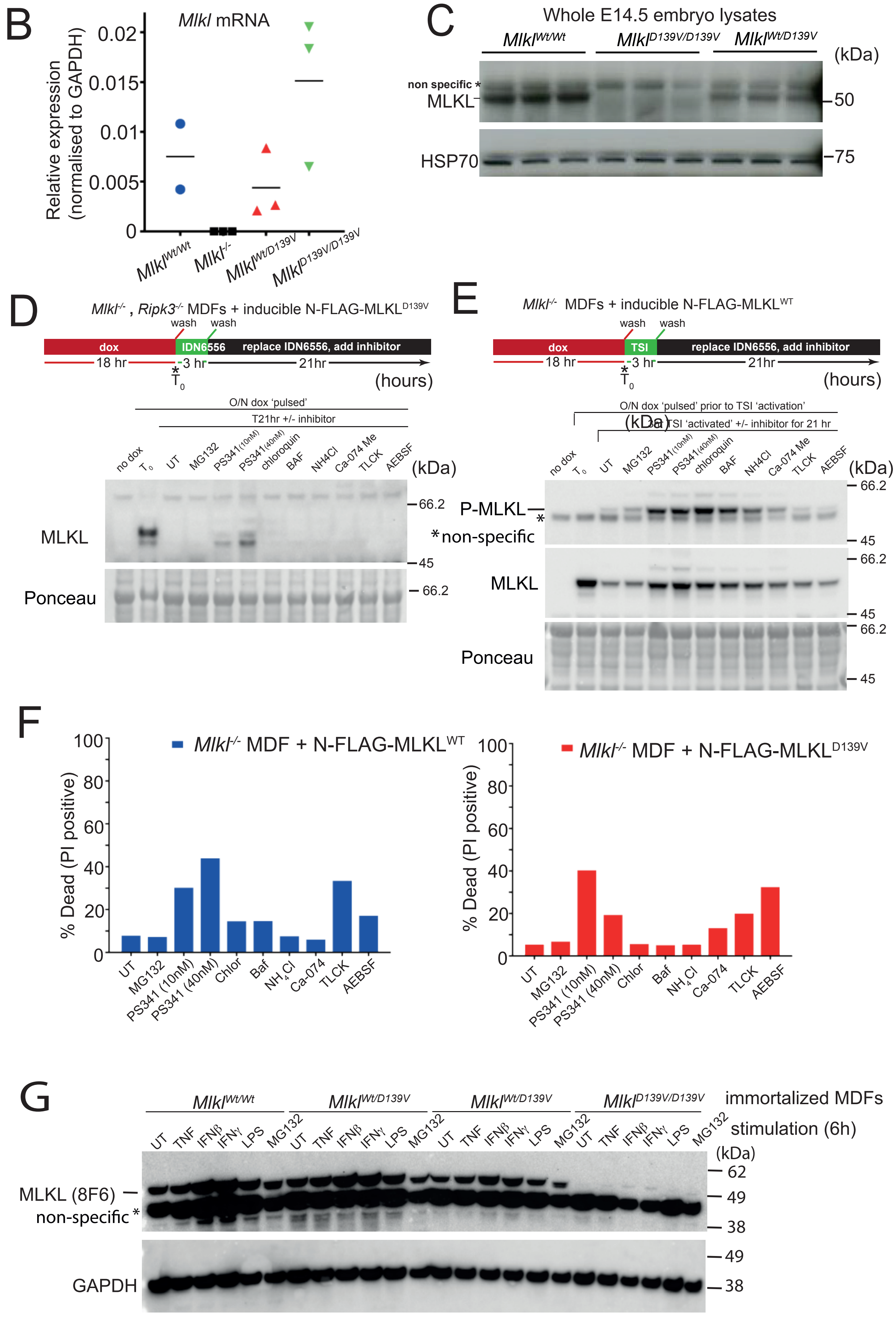

Hildebrand and Kauppi *et al*, Supplementary Figure 4

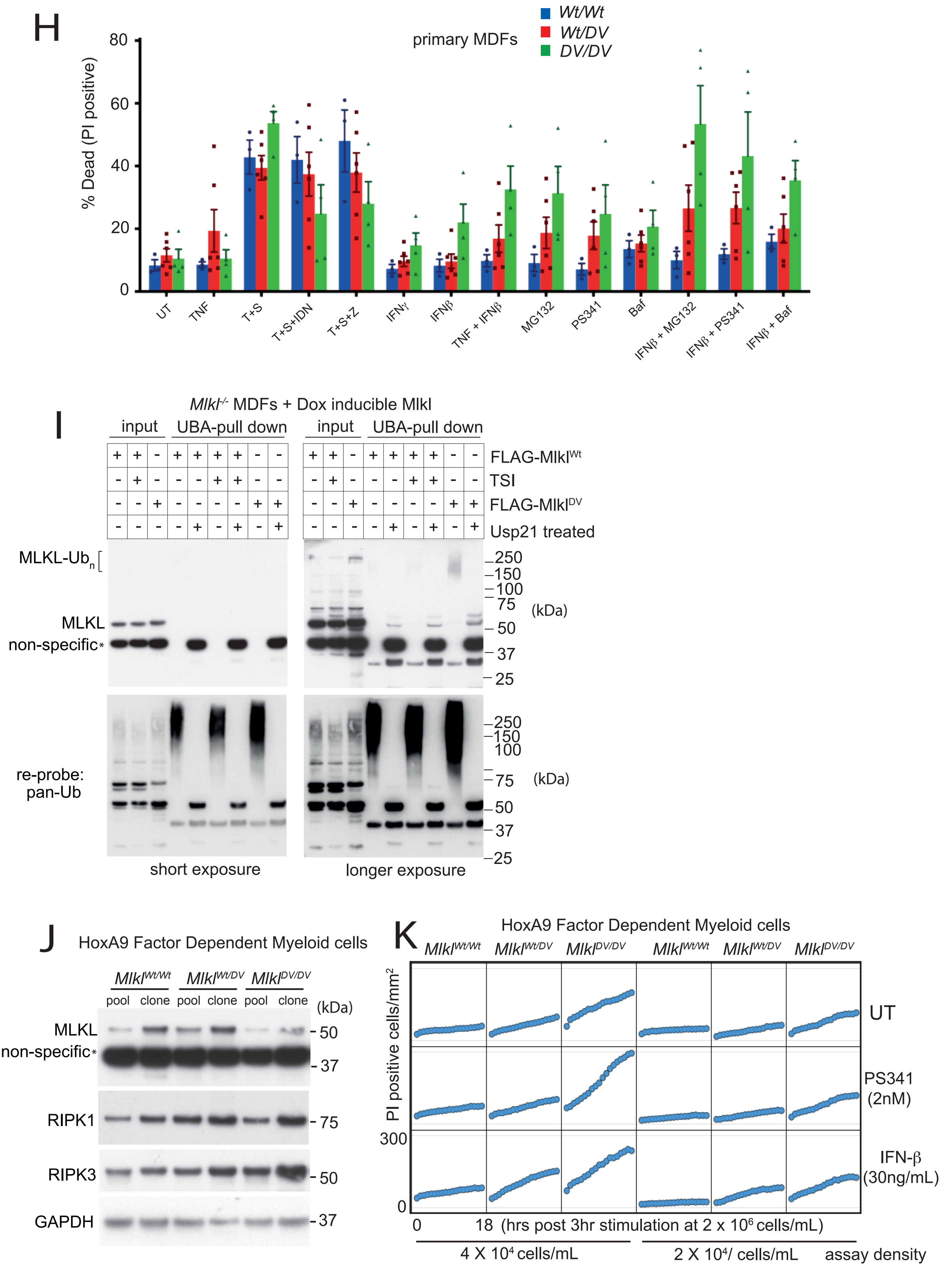

Hildebrand and Kauppi *et al*, Supplementary Figure 4

Hildebrand and Kauppi *et al*, Supplementary Figure 4. (A) Two biologically independent immortalised MDF lines derived from pups of the indicated genotypes were stimulated as indicated. PI positive cells were quantified over time using an IncuCyte automated cell imager. **(B)** Mouse *Mlkl* mRNA levels in MDFs quantified using TaqMan probes. n = 2 for Wt/Wt and n = 3 for all other genotypes. Error bars indicate mean ± range for Wt/Wt and mean ± SD for all other genotypes. **(C)** E14.5 whole embryo lysates from 3 pups per genotype were probed by western blot for relative MLKL protein levels. **(D)** *Mlkl*$^{-/-}$, *RIPK3*$^{-/-}$ MDFs were stably transduced with doxycycline-inducible FLAG-MLKL^{D139V}, **(E)** and *Mlkl*$^{-/-}$ MDFs were stably transduced with doxycycline-inducible FLAG-MLKL^{WT} MLKL protein stability after doxycycline and in the presence of indicated compounds. **(F)** Viability of cells following 21 hr incubation with inhibitors used in **Fig 4D** and **Supp. Fig. 4D,E. -E.** Representative of 3 similar experiments. **(G)** MDFs were treated as indicated for 6 hours. Whole cell lysates were analysed by western blot for levels of MLKL. **(H)** Primary MDFs were isolated from *Mlkl*$^{Wt/Wt}$, *Mlkl*$^{Wt/D139V}$, *Mlkl*$^{D139V/D139V}$ mouse pups and stimulated as indicated for 21hrs for quantification of PI positive cells using flow cytometry. N = 3, 6 and 4 independent experiments respectively and data plotted as mean ± SEM. **(I)** MDFs expressing FLAG-MLKL^{WT} and FLAG-MLKL^{D139V} were stimulated with doxycycline +/- TSI for 5 hrs as indicated and lysates subject to UBA-GST enrichment +/- digestion with Usp21 deubiquitinase. **(J)** HOXA9-immortalised factor dependent myeloid cell pools or clonal populations containing equal numbers of viable cells were lysed and probed by western blot for the indicated proteins or **(K)** incubated in the presence of indicated agents for enumeration of PI permeant cells over time using an IncuCyte automated cell imager. All images included are representative of at least 3 independent experiments with similar results.

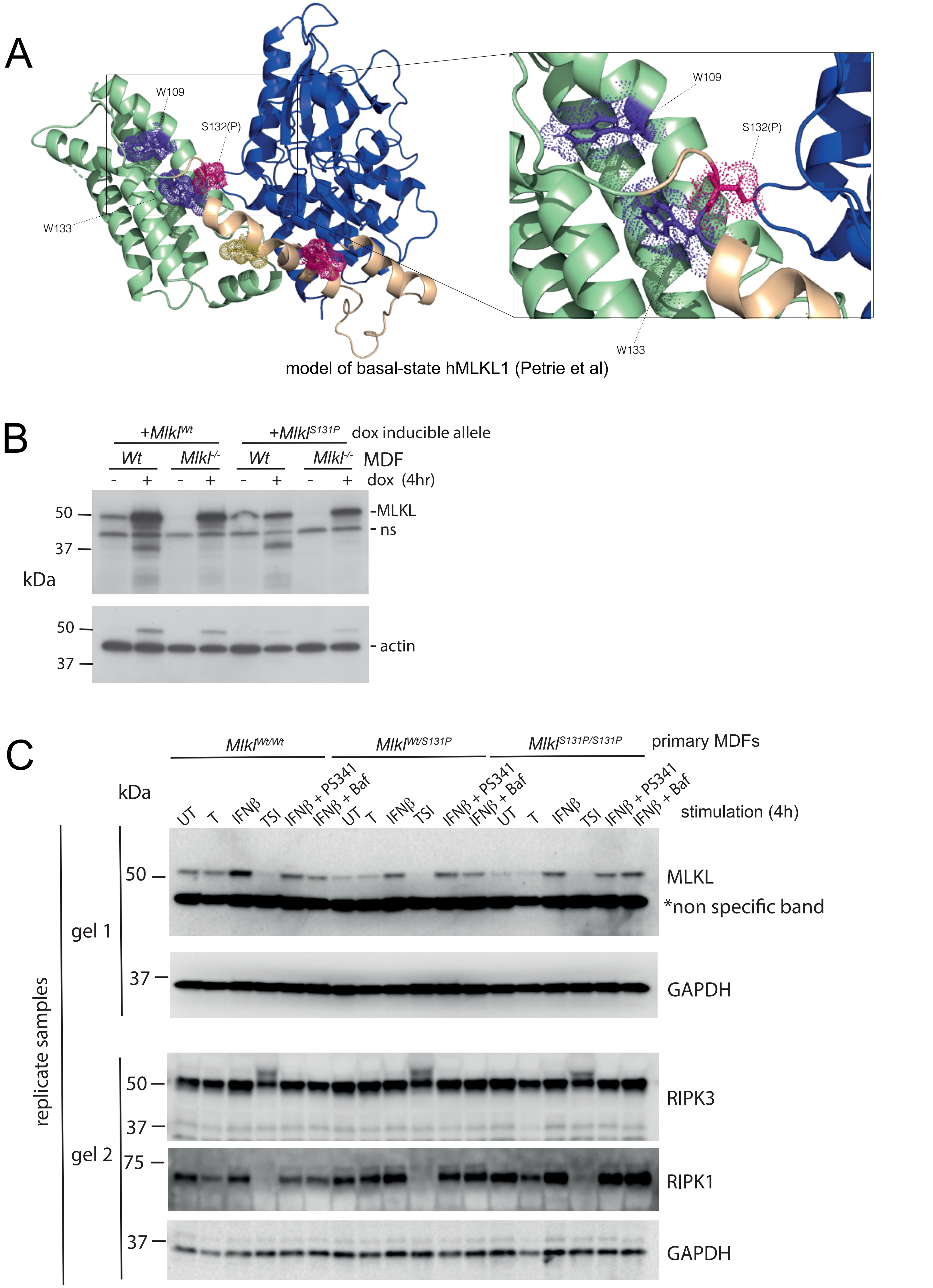

Hildebrand and Kauppi *et al*, Supplementary Figure 5

D

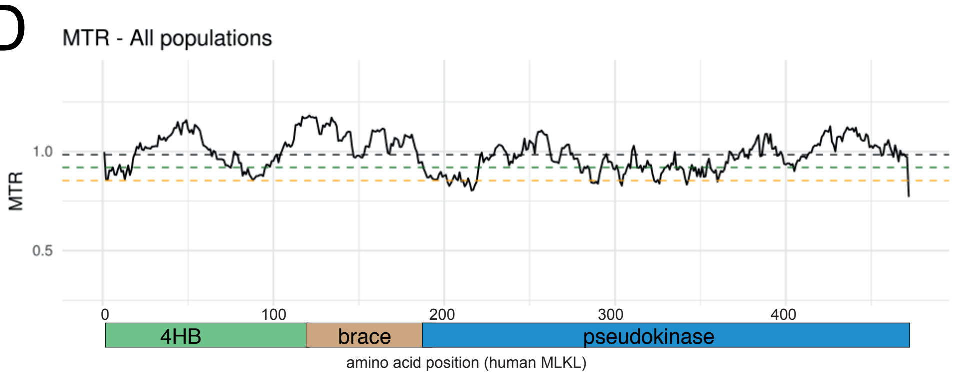

Hildebrand and Kauppi *et al*, Supplementary Figure 5. (A) A proline in position 132 of human MLKL is predicted to significantly impact the conformation of the immediately adjacent W133 (brace helix) and in turn, the closely situated W109 (4 helix bundle). **(B)** MDFs stably transduced with doxycycline inducible constructs expressing mouse MLKL^{S131P} were analysed by western blot for MLKL levels after 4 hrs dox induction. **(C)** Primary MDFs were isolated from $\mathit{Mlkl}^{Wt/Wt}$, $\mathit{Mlkl}^{Wt/S131P}$ or $\mathit{Mlk}^{S131P/S131P}$ mouse pups and were treated as indicated for 4 hours. Whole cell lysates were analysed by western blot for levels of MLKL. **(B)** and **(C)** representative of at least 3 independent experiments with similar results. **(D)** Missense Tolerance Ratio (MTR) distribution for human MLKL using gnomAD exome data.

Supplementary Table 1. Outcome of *Mlkl*$^{Wt/D139}$ x *Mlkl*$^{Wt/D139}$ cross

Age	Number of litters	Number of mice	*Mlkl*$^{Wt/Wt}$	*Mlkl*$^{Wt/D139V}$	*Mlkl*$^{D139V/D139V}$
E13.5	1	8	2(2)	3(4)	3(2)
E14.5	22	156	58(39)	70(78)	28(39)
E16.5	3	27	5(7)	19(14)	3(7)
E17.5	2	23	7(6)	9(12)	8(6)
E18.5	5	39	7(10)	17(20)	13(10)
P21	12	45	15(11)	30(23)	0(11)

Embryos from matings between *Mlkl*$^{Wt/D139V}$ and *Mlkl*$^{Wt/D139V}$ mice were genotyped at the gestational (E) or postnatal (P) age indicated (days). Observed numbers of *Mlkl*$^{Wt/Wt}$, *Mlkl*$^{Wt/D139V}$ and *Mlkl*$^{D139V/D139V}$ embryos tabulated with numbers expected from Mendelian inheritance (in the absence of lethality) indicated in parentheses.

Supplementary Table 2. Outcome of *Mlkl*$^{Wt/D139}$ x *Mlkl*$^{null/null}$ cross

Age	Number of litters	Number of mice	*Mlkl*$^{Wt/null}$	*Mlkl*$^{D139V/null}$
P21	8	40	19 (20)	21 (20)

Surviving progeny from matings between *Mlkl*$^{null/null}$ and *Mlkl*$^{Wt/D139V}$ mice were genotyped at postnatal day 21. Observed numbers are tabulated, with numbers expected from mendelian inheritance in the absence of lethality indicated in parentheses.

Supplementary Table 3. Outcome of CRISPR-*Mlkl*$^{Wt/D139V}$ x CRISPR-*Mlkl*$^{Wt/D139V}$ cross

Age	Number of litters	Number of mice	*Mlkl*$^{Wt/Wt}$	*Mlkl*$^{Wt/D139V}$	*Mlkl*$^{D139V/D139V}$
P21	7	36	12 (9)	24 (18)	0 (9)

Surviving progeny from matings between CRISPR-induced Mlkl$^{Wt/D139V}$ mice were genotyped at postnatal day 21. Observed numbers are tabulated, with numbers expected from mendelian inheritance in the absence of lethality indicated in parentheses.

Supplementary Table 4. *hMLKL* brace helix variants - MAFs in CRMO vs Healthy Controls

Feature	human *MLKL* SNP		
	R146Q- rs34515646	*S132P- rs35589326*	*G202*V- rs144526386*
1000 genomes- EU MAF (n)	0.0179 (503)	0.0149 (503)	0.0209 (503)
U.Iowa CRMO cohort Total MAF (n)	0.0273 (128)	0.0234 (128)	0.0234 (128)
U.Iowa CRMO cohort EU MAF (n)	0.0347 (101)	0.0198 (101)	0.0198 (101)
U.Iowa CRMO TOTAL vs 1000 genomes Total MAF	p= 0.0009	p= 0.0329	p= 0.057
U.Iowa CRMO EU vs 1000 genomes EU MAF	p= 0.1687	p= 0.5423	p= 0.99
SIFT Score (classification)	MLKL1- 0.569 (TOLERATED)<br>MLKL2- 0.536 (TOLERATED)	MLKL1- 0.25 (TOLERATED)<br>MLKL2- 0 (DELETERIOUS)	MLKL2- 0.069 (TOLERATED)
POLYPHEN-2 Score (classification)	MLKL1- 0.114<br>(BENIGN)	MLKL1- 0.996<br>(PROBABLY DAMAGING)	n/a

n - number of unrelated individuals sequenced
MAF - Minor Allele Frequency -count
**alternate transcript*
p - 2-tailed fisher's exact p-value by comparing the allele counts in cases and controls

Supplementary Table 5. *hMLKL* brace helix MAFs in AS, GB and SAPHO vs Healthy Controls

Disease	*MLKL* SNP	Disease MAF (n)	matched healthy control MAF (n)	*p* value
Ankylosing Spondylitis	*R146Q*	*0.0274 (8244)* imputed	*0.0255(14542)*	*0.227*
	S132P	*0.017 (8244)* genotyped	*0.0165 (14542)*	*0.699*
	*G202*V*	*0.0144 (8244)* genotyped	*0.0155 (14542)*	*0.385*
Guillain-Barre syndrome	*R146Q*	*0.0084 (178)* imputed	0.0255(14542)	*0.328*
	S132P	*N/A (INFO score <0.6)* imputed	0.0165 (14542)	*N/A*
	*G202*V*	*0.0112 (178)*	0.0155 (14542)	*0.665*
SAPHO	*R146Q*	*0.0227 (22)*	0.0052 (2,504)	*0.960*
	S132P	*0.0227 (22)*	0.0088 (2,504)	*0.327*
	*G202*V*	*N/A*	0.0102 (2,504)	*N/A*

n - number of unrelated invividuals sequenced
MAF - Minor Allele Frequency-count
N/A - not available
p = chi-square test (with Yates' continuity correction) by comparing the allele counts in cases and controls
