# Supplementary material for: A missense mutation in the MLKL brace region promotes lethal neonatal inflammation and hematopoietic dysfunction
Source: Nat Commun. 2020 Jun 19;11:3150. doi: 10.1038/s41467-020-16819-z (PMC7305203; doi:10.1038/s41467-020-16819-z)
Supplement: Supplementary file 2 — Reporting Summary [file 41467_2020_16819_MOESM2_ESM.pdf]

nature research

Corresponding author(s): Dr Joanne Hildebrand
Prof. Warren Alexander
Prof. John Silke

Last updated by author(s): May 12, 2020

Reporting Summary

Nature Research wishes to improve the reproducibility of the work that we publish. This form provides structure for consistency and transparency in reporting. For further information on Nature Research policies, see Authors & Referees and the Editorial Policy Checklist.

Statistics

For all statistical analyses, confirm that the following items are present in the figure legend, table legend, main text, or Methods section.

n/a	Confirmed	
☐	☒	The exact sample size (*n*) for each experimental group/condition, given as a discrete number and unit of measurement
☐	☒	A statement on whether measurements were taken from distinct samples or whether the same sample was measured repeatedly
☐	☒	The statistical test(s) used AND whether they are one- or two-sided<br>*Only common tests should be described solely by name; describe more complex techniques in the Methods section.*
☐	☒	A description of all covariates tested
☐	☒	A description of any assumptions or corrections, such as tests of normality and adjustment for multiple comparisons
☐	☒	A full description of the statistical parameters including central tendency (e.g. means) or other basic estimates (e.g. regression coefficient) AND variation (e.g. standard deviation) or associated estimates of uncertainty (e.g. confidence intervals)
☒	☐	For null hypothesis testing, the test statistic (e.g. *F*, *t*, *r*) with confidence intervals, effect sizes, degrees of freedom and *P* value noted<br>*Give P values as exact values whenever suitable.*
☒	☐	For Bayesian analysis, information on the choice of priors and Markov chain Monte Carlo settings
☒	☐	For hierarchical and complex designs, identification of the appropriate level for tests and full reporting of outcomes
☒	☐	Estimates of effect sizes (e.g. Cohen's *d*, Pearson's *r*), indicating how they were calculated

Our web collection on statistics for biologists contains articles on many of the points above.

Software and code

Policy information about availability of computer code

Data collection	gnomAD – https://gnomad.broadinstitute.org/<br>http://asia.ensembl.org<br>OrthoDB - https://www.orthodb.org<br>CADD - https://cadd.gs.washington.edu/<br>Clustal Omega - https://www.ebi.ac.uk/Tools/msa/clustalo/<br>WEBLOGO - https://weblogo.berkeley.edu/logo.cgi<br>Missense Tolerance Ratio (MTR) Gene Viewer - http://biosig.unimelb.edu.au/mtr-viewer<br>UK biobank - https://www.ukbiobank.ac.uk<br>Understanding Society - https://www.understandingsociety.ac.uk/<br>gnomAD – https://gnomad.broadinstitute.org/<br>http://asia.ensembl.org<br>OrthoDB - https://www.orthodb.org<br>CADD - https://cadd.gs.washington.edu/<br>Clustal Omega - https://www.ebi.ac.uk/Tools/msa/clustalo/<br>WEBLOGO - https://weblogo.berkeley.edu/logo.cgi<br>Missense Tolerance Ratio (MTR) Gene Viewer - http://biosig.unimelb.edu.au/mtr-viewer<br>UK biobank - https://www.ukbiobank.ac.uk<br>Understanding Society - https://www.understandingsociety.ac.uk/
Data analysis	IncuCyte® S3 Software (V2018B), FlowJo V10.2, Local Thickness plugin in ImageJ (http://www.optinav.info/Local_Thickness.htm), SDS version 2.3 (Applied Biosystems), GATK<br>HaplotypeCaller, Broad Institute, LASER V2.0

For manuscripts utilizing custom algorithms or software that are central to the research but not yet described in published literature, software must be made available to editors/reviewers. We strongly encourage code deposition in a community repository (e.g. GitHub). See the Nature Research guidelines for submitting code & software for further information.

Data

Policy information about availability of data

All manuscripts must include a data availability statement. This statement should provide the following information, where applicable:

- Accession codes, unique identifiers, or web links for publicly available datasets
- A list of figures that have associated raw data
- A description of any restrictions on data availability

We provide a full description of the publicly available web resources and the sources of human genomic data. Human genomic data generated as part of this study is controlled access at the time of publication due to ongoing studies. MLKL gene variants in CRMO can be accessed from the European Genome-Phenome Archive under accession code indicated in manuscript. hMLKL variants have been posted to ClinVar. MLKL brace SNP MAF data from gnomAD, the NIH and the UK biobank can be accessed via the following links; [https://gnomad.broadinstitute.org/gene/ENSG00000168404?dataset=gnomad_r2_1], [http://asia.ensembl.org/Homo_sapiens/Gene/Variation_Gene/Table?db=core;g=ENSG00000168404;r=16:74671855-74700960] and [http://biobank.ndph.ox.ac.uk/showcase/gsearch.cgi] and entering relevant rs numbers. The source data underlying Figs. 1a, d, e-g, 2a–f, 3a-k, 4a-f, 5c-d and Supplementary Figs. 1a-b, 2a-h, 3a, c, d-f, 4b, d- j are provided as a Source Data file.

Field-specific reporting

Please select the one below that is the best fit for your research. If you are not sure, read the appropriate sections before making your selection.

☒ Life sciences ☐ Behavioural & social sciences ☐ Ecological, evolutionary & environmental sciences

For a reference copy of the document with all sections, see nature.com/documents/nr-reporting-summary-flat.pdf

Life sciences study design

All studies must disclose on these points even when the disclosure is negative.

Sample size	No sample size calculations were performed. Sample sizes were determined by availability of biological replicates (sequenced human individuals, individual animals of appropraite genotypes or cell lines derived from individual animals) and processing capacity and are consistent with field-norms. Sample numbers are stated in each figure legend.
Data exclusions	No data were excluded from these analyses.
Replication	All experimental findings were replicated both through independent experimental repeats perfopmred on different days, and/or through the use of independent biological replicates. All attempts at replication were successful.
Randomization	All mice, cells derived from individual mice and cell lines were randomly allocated to experimental groups.
Blinding	Investigators were blinded to group allocation during measurement and analyses

Reporting for specific materials, systems and methods

We require information from authors about some types of materials, experimental systems and methods used in many studies. Here, indicate whether each material, system or method listed is relevant to your study. If you are not sure if a list item applies to your research, read the appropriate section before selecting a response.

Materials & experimental systems

n/a	Involved in the study	
☐	☒	Antibodies
☐	☒	Eukaryotic cell lines
☒	☐	Palaeontology
☐	☒	Animals and other organisms
☐	☒	Human research participants
☒	☐	Clinical data

Methods

n/a	Involved in the study	
☒	☐	ChIP-seq
☐	☒	Flow cytometry
☒	☐	MRI-based neuroimaging

Antibodies

Antibodies used

anti mRIPK3; WEHI antibody facility Monoclonal # 8G7 - validated alongside RIPK3-/- cell lines in Fig. 1F
anti-mMLKL 8F6 (residues 1-30); WEHI antibody facility Monoclonal # 8F6 - validated alongside MLKL-/- cell lines in Fig. 4B
anti-mMLKL 3H1 (brace region) WEHI antibody facility Monoclonal # 3H1 - published extensively since 2013 and available commercially through Merck-Millipore MABC604
anti-Pro Caspase 8 Cell Signaling Technology Cat#4927- published extensively, validation available online
anti-GAPDH Cell Signaling Technology Cat#2113- published extensively, validation available online

Anti-Cleaved caspase 3 Cell Signaling Technology Cat#9661- published extensively, validation available online
Anti-CD45 BD Pharmingen Cat#550539- published extensively, validation available online
anti-phospho mMLKL Abcam Cat#ab196436- published extensively, validation available online
anti-actin Abcam Cat#ab5694- published extensively, validation available online
anti-VDAC Millipore Cat#AB10527- published extensively, validation available online
CD117 PerCPCy5.5 clone 2.4G2 BD Cat#560557- published extensively, validation available online
CD117 BV711 BD clone 2B8 BD Cat# 563160- published extensively, validation available online
Sca1 PE BD Cat# 553108- published extensively, validation available online
CD135 PE clone A2F10 Biolegend Cat# 135305- published extensively, validation available online
CD150 BV421 clone TC15-12F12.2 Biolegend Cat#115926- published extensively, validation available online
CD48 PECy7 clone HM48-1 eBioscience Cat# 11-0481-85- published extensively, validation available online
CD16/32 PerCPCy5.5 clone 2.4G2 BD Cat# 560540- published extensively, validation available online
CD16/32 PerCPe710 eBioscience Cat# 46-0161-80- published extensively, validation available online
CD105 PE clone MJ718 eBioscience Cat# 12-1051-83- published extensively, validation available online
CD9 AlexaFluor647 clone KMC8 BD Cat# 564233- published extensively, validation available online
CD41 BV605 Biolegend Cat# 133921- published extensively, validation available online
Ly5.2 PerCPCy5.5 BD Cat# 5148766- published extensively, validation available online
Ly5.2 FITC clone 104 BD Cat#553772- published extensively, validation available online
Ly5.1 biotin eBioscience Cat#13-0453-85- published extensively, validation available online
Streptavidin PECy7 BD Cat#557598- published extensively, validation available online
CD2 clone RM2.1, CD4 clone GK1.5, CD8 clone 53.6.7, Gr1 clone RB6-8C5, F4/80, CD19 clone 1D3
B220 clone RA3-6B2, Ly6G clone 1A8. Ter119, AnnexinV FITC, Sca1 A594 clone E13-161-7, CD9 FITC
and CD16/32 FITC were all produced in-house by the WEHI antibody facility. All in-house antibodies are which validates all reagents using marker positive and marker negative calls alongside isotype control antibodies. All antibodies used at dilutions between 1:1000 and 1:10,000 for western blot analyses, and between 1:50 and 1:250 for FACs staining.

Validation

see above, all validation information for commercial antibodies available by searching for catalogue numbers of manufacturer's website

Eukaryotic cell lines

Policy information about cell lines

Cell line source(s)

All cell lines used were derived by the authors themselves from mice generated by the Walter and Eliza Hall Institute of Medical research

Authentication

Genotype and species identity of all cell lines were authenticated in house using PCR based methods.

Mycoplasma contamination

All cell lines are routinely tested for mycoplasma contamination using PCR-based methods. Cell lines used in this study all tested negative for mycoplasma

Commonly misidentified lines
(See ICLAC register)

none were used

Animals and other organisms

Policy information about studies involving animals; ARRIVE guidelines recommended for reporting animal research

Laboratory animals

Both male and female mice from 129/SV and C57BL/6 strains were used, strain and age of mice indicated for each experiment. Sex was not recorded for animals samples at E19.5, P2 and P3. Precise age and sex of all adult mice used are reported in 'Methods' where relevant.

Wild animals

no wild animals were used

Field-collected samples

no field-collected samples were used

Ethics oversight

The WEHI Animal Ethics Committee approved all experiments in accordance with the
NHMRC Australian code for the care and use of animals for scientific purposes.

Note that full information on the approval of the study protocol must also be provided in the manuscript.

Human research participants

Policy information about studies involving human research participants

Population characteristics

All human genomic data was derived from patients and healthy controls recruited for previously published studies. Details of patient characteristics, recruitment and ethics oversight can be found in the following publications;
Cox, A. J. et al. Recessive coding and regulatory mutations in FBLIM1 underlie the pathogenesis of chronic recurrent multifocal osteomyelitis (CRMO). PLoS One 12, e0169687, doi:10.1371/journal.pone.0169687 (2017).
Blum, S. et al. Genome-wide association study in Guillain-Barre syndrome. J Neuroimmunol 323, 109-114, doi:10.1016/j.jneuroim.2018.07.016 (2018).
Lek, M. et al. Analysis of protein-coding genetic variation in 60,706 humans. Nature 536, 285-291, doi:10.1038/nature19057 (2016).

	University of Essex. Institute for Social and Economic Research, NatCen Social Research, Kantar Public. (2018). Understanding Society: Waves 1-8, 2009-2017 and Harmonised BHPS: Waves 1-18, 1991-2009. [data collection]. 11th Edition. UK Data Service. Reveille, J. D. et al. HLA class I and II alleles in susceptibility to ankylosing spondylitis. Annals of the rheumatic diseases 78, 66-73, doi:10.1136/annrheumdis-2018-213779 (2019).
Recruitment	No patients were recruited for this study specifically.
Ethics oversight	All genomic data was collected with the approval of human ethics review boards of all Institutes that participated in human genetics studies; University of Iowa Carver College of Medicine, Queensland University of Technology, Australian National University, Shanghai Renji Hospital,<br>JiaoTong University of Shanghai, The Hospital for Sick Children and the University of Toronto, University of Sydney, Australian Institute of Sport, University of<br>Freiburg, Princess Alexandra Hospital, Memorial Hermann Texas Medical Centre, The University of Queensland, Oregon Health and Science University), Groupe<br>Française d'Etude Génétique des Spondylarthrites (GFEGS) and the University of Oxford.

Note that full information on the approval of the study protocol must also be provided in the manuscript.

Flow Cytometry

Plots

Confirm that:

- [ ] The axis labels state the marker and fluorochrome used (e.g. CD4-FITC).
- [ ] The axis scales are clearly visible. Include numbers along axes only for bottom left plot of group (a 'group' is an analysis of identical markers).
- [ ] All plots are contour plots with outliers or pseudocolor plots.
- [ ] A numerical value for number of cells or percentage (with statistics) is provided.

Methodology

Sample preparation	No Flow cytometry plots are provided as data, with the exception of example plots used to describe gating strategy. Information pertaining to staining, instrumentation, software and gating strategies are available in previously published papers<br>Murphy, J. M. et al. The pseudokinase MLKL mediates necroptosis via a molecular switch mechanism. Immunity 39, 443-453, doi:10.1016/j.immuni.2013.06.018 (2013).<br>Kauppi, M. et al. Point mutation in the gene encoding p300 suppresses thrombocytopenia in Mpl-/- mice. Blood 112, 3148-3153, doi:10.1182/blood-2007-10-119677 (2008).
Instrument	LSRI, LSRII or Fortessa1 (BD Biosciences)
Software	FlowJo 10.2
Cell population abundance	n/a
Gating strategy	provided as Supplemental Figure 3B

- [x] Tick this box to confirm that a figure exemplifying the gating strategy is provided in the Supplementary Information.
